# Spatiotemporal Distribution Patterns and Conservation Priorities of Gymnosperms With Different Leaf Shapes in China Under Climate Change

**DOI:** 10.1002/ece3.71980

**Published:** 2025-08-14

**Authors:** Jinyi Fu, Wenjie Song, Chuncheng Wang, Xiaolong Jiang, Xiangbao Shen, Rong Yi

**Affiliations:** ^1^ College of Forestry, The Laboratory of Forestry Genetics, Central South University of Forestry and Technology Changsha People's Republic of China; ^2^ College of Landscape Architecture, Central South University of Forestry and Technology Changsha People's Republic of China

**Keywords:** climate change, conservation gap, geographic distribution, gymnosperms, multi‐species distribution modeling

## Abstract

Leaf morphology is one of the important indicators for studying the response of plants to climate change. Gymnosperms play a crucial role in maintaining biodiversity and ecosystem stability in China. However, the geographical and altitudinal distribution patterns of gymnosperms with different leaf morphologies in China in response to climate change are not yet fully understood. This study utilized occurrence data for 71 rare gymnosperm species (including varieties) and 15 environmental variables to model the contemporary geographical distribution for the 2070s and the 2090s under two shared socioeconomic pathway scenarios (SSP2‐4.5 and SSP5‐8.5). Gymnosperm species were classified into five groups based on their leaf shapes (needle‐shaped, scale‐shaped, lanceolate‐shaped, fan‐shaped, and strip‐shaped), and the analysis revealed that the primary climatic variable driving ecological niche differences among these groups was Bio15 (precipitation seasonality). Lanceolate‐leaved gymnosperms exhibited an expansionary trend, whereas other groups generally showed range reductions under future climatic scenarios. The results indicated that approximately half of the gymnosperm species will experience notable range contractions and gradual migration to higher altitudes in northwestern regions from the present to the 2090s. Hotspots for species richness were identified in the eastern Yunnan‐Guizhou Plateau, the Nanling Mountains, and the eastern Zhejiang‐Fujian Hills. However, these hotspots showed limited overlap with existing nature reserves in China. The threat status of some species will be severely upgraded from vulnerable to critically endangered, such as *Abies recurvata*, highlighting the urgent need for enhanced conservation efforts. This study enhances understanding of the future distribution patterns of China's gymnosperms and provides valuable insights for developing targeted protection and conservation strategies.

## Graphical abstract

This study modeled the spatiotemporal distribution patterns of 71 rare gymnosperm species (including varieties) with five different leaf morphologies in China in response to climate change and found that approximately half of the species will experience significant range contractions and migrate to higher altitudes toward the northwest. Lanceolate‐leaved gymnosperms show range expansions compared to other groups. The overlap between the regions of high species richness and existing national nature reserves remains limited, posing the need for targeted conservation strategies.

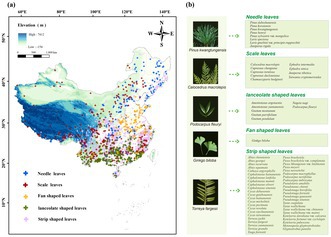

## 1 Introduction

Rapid climate change is recognized as a significant factor in influencing plant growth, geographical distribution, community composition, and species diversity (Pereira et al. 2010; Zhang et al. 2021; Guo et al. 2024). The Sixth Assessment Report of the Intergovernmental Panel on Climate Change (IPCC) projects that the global average annual temperature will increase by 1.0°C to 5.7°C by the end of the 21st century, posing severe challenges to ecosystems and biological communities (IPCC 2021). In China, approximately 11% of the country's 35,784 higher plant species are classified as threatened by climate change, yet only 71.5% of near‐threatened species fall within the boundaries of nature reserves (Qin et al. 2017; Liu et al. 2023). In addition to climate change, topographic succession, soil heterogeneity, and human activities exert a significant impact on species distribution (Pecl et al. 2017; Hu et al. 2020). Variations in elevation and slope can create microclimates and geomorphological differences that affect plant growth and distribution. Soil type and humidity significantly modulate water absorption and nutrient production, further shaping plant communities. However, alterations in land‐use patterns—driven by agricultural expansion, urbanization, and industrial activities—along with the overexploitation of plant resources, contribute to habitat loss, fragmentation, and declining species diversity (Harrison and Bardgett 2010; Pepin et al. 2022; Dad et al. 2023). A comprehensive approach to species diversity and conservation management must account for spatial and temporal scales and integrate multiple environmental factors. This holistic perspective is essential for developing effective strategies to mitigate biodiversity loss and ensure the resilience of plant communities under future climate conditions.

Gymnosperms represent a pivotal evolutionary group, marking the transition from spore reproduction to seed reproduction in higher plants. Originating in the Middle Devonian period approximately 385 million years ago, gymnosperms flourished during the Mesozoic era but gradually declined since the Tertiary period due to climate change and interspecific competition with angiosperms (Wang and Ran 2014; Wu et al. 2023). The complex topography and relatively stable climatic conditions of subtropical mountainous regions in China have provided crucial refuges and expansion areas for gymnosperms during the Tertiary and Quaternary periods (Song et al. 2024; Tang et al. 2018). Today, China harbors approximately one‐fifth of the world's gymnosperm species, including nearly 100 endemic species (Huang et al. 2015; Yang 2015). The diversity of gymnosperms in China follows a distinct latitudinal pattern, with higher species richness in the south and lower diversity in the north. Species diversity centers are concentrated in the Hengduan Mountains and adjacent mountainous areas (Lü et al. 2018; Yang et al. 2017). As essential indicators of ancient flora and fauna, gymnosperms provide valuable insights into the relationships among extinct species, the evolution of terrestrial plants, and historical changes in biodiversity. They play an irreplaceable role in maintaining biodiversity, stabilizing ecosystems, and promoting sustainability (Yao et al. 2011). However, global warming, habitat degradation, and human activities have severely impacted the suitable habitats of gymnosperms in China, with only approximately 60 threatened species currently protected within nature reserves (Wu 2020; Xie et al. 2021). This underscores the urgent need to investigate the geographical distribution patterns of gymnosperms and identify biodiversity migration hotspots under future climate change scenarios to inform effective conservation strategies.

Leaves, as essential nutritional organs of plants, have developed distinct morphological and physiological traits through long‐term adaptation to climate changes. The spatial and temporal distribution patterns of plants with varying leaf shapes are closely linked to climatic factors (Li et al. 2020). For example, oaks tend to have round leaves in dry habitats and lanceolate leaves in warm and humid environments (Martín‐Sánchez et al. 2024). Gymnosperms, known for their sensitivity to climate change, have predominantly evolved needle‐shaped, scale‐shaped, and strip‐shaped leaves as adaptive responses to dry and cold conditions. Pinaceae and Cupressaceae species, characterized by needle‐like and scale‐like leaves, thrive across various climate zones and soil types, primarily in mountainous regions of China (Xie et al. 2022); Cycadaceae species, distinguished by their striped leaves, prefer hot, humid environments and are commonly found in tropical forests, evergreen broad‐leaved forests, and secondary forests (Zheng et al. 2017); Podocarpaceae, Taxaceae, and certain Cupressaceae species with scale‐like and lanceolate leaves are concentrated in cool, moist environments within coniferous or mixed forests, often flourishing in diverse soil conditions (Wu et al. 2022). Some Ephedraceae species with scale‐shaped leaves demonstrate exceptional drought resistance, salt tolerance, and the ability to survive in infertile conditions, enabling them to establish extensive habitats in dry hillsides, wastelands, and grasslands (Liu et al. 2023); fan‐shaped leaf plants, such as *Ginkgo biloba* , thrive in areas with abundant sunlight, with wild populations only occurring in certain mountainous regions such as Tianmu Mountain and Dabie Mountain, as well as the middle and lower reaches of the Yangtze River (Guo et al. 2019). Examining the distribution patterns of plants with different leaf morphologies and their responses to climate change provides important insights into their long‐term survival strategies and community composition (Laughlin 2024). Although previous studies have primarily focused on the geographic distribution of individual or representative gymnosperm species, limited attention has been given to the dynamic distribution patterns of rare gymnosperms with different leaf shapes across spatial and temporal scales. This gap in research hinders a comprehensive understanding of gymnosperm diversity, evolution, and the formation of conservation strategies.

Species distribution models (SDMs) integrate species occurrence data with environmental variables using statistical and machine‐learning algorithms. These models are extensively applied in biogeography and conservation biology to evaluate the impacts of climate change on species distributions, identify regions of high biodiversity value, and delineate suitable areas for the conservation translocation of threatened species (Wang et al. 2023). Biomod2 is an ensemble modeling platform for species distribution predictions, developed in the R programming language. It integrates multiple models to simulate species distribution ranges and employs consensus methods, such as boundary values, commonality, or probability weighting, to reduce biases associated with single‐model approaches and enhance prediction accuracy (Buisson et al. 2010; Thuiller et al. 2009). To date, the Biomod2 ensemble models have been extensively applied to predict the potential geographic distributions of a wide range of species. In this study, the spatiotemporal distribution of 71 rare gymnosperm species (including varieties) in China was analyzed using geographic distribution data and various environmental variables. The Biomod2 species distribution model was applied to project distributions for the current period, the 2070s, and the 2090s. The objectives of this study are to (1) identify the dominant environmental factors influencing species richness and ecological niche differentiation among gymnosperms with diverse leaf morphologies; (2) characterize spatiotemporal trends in species range shifts and variations in species richness; and (3) evaluate changes in species endangerment levels and prioritize gymnosperms facing increased threats.

## 2 Material and Methods

### 2.1 Species Distribution Data and Classification

**FIGURE 1:**
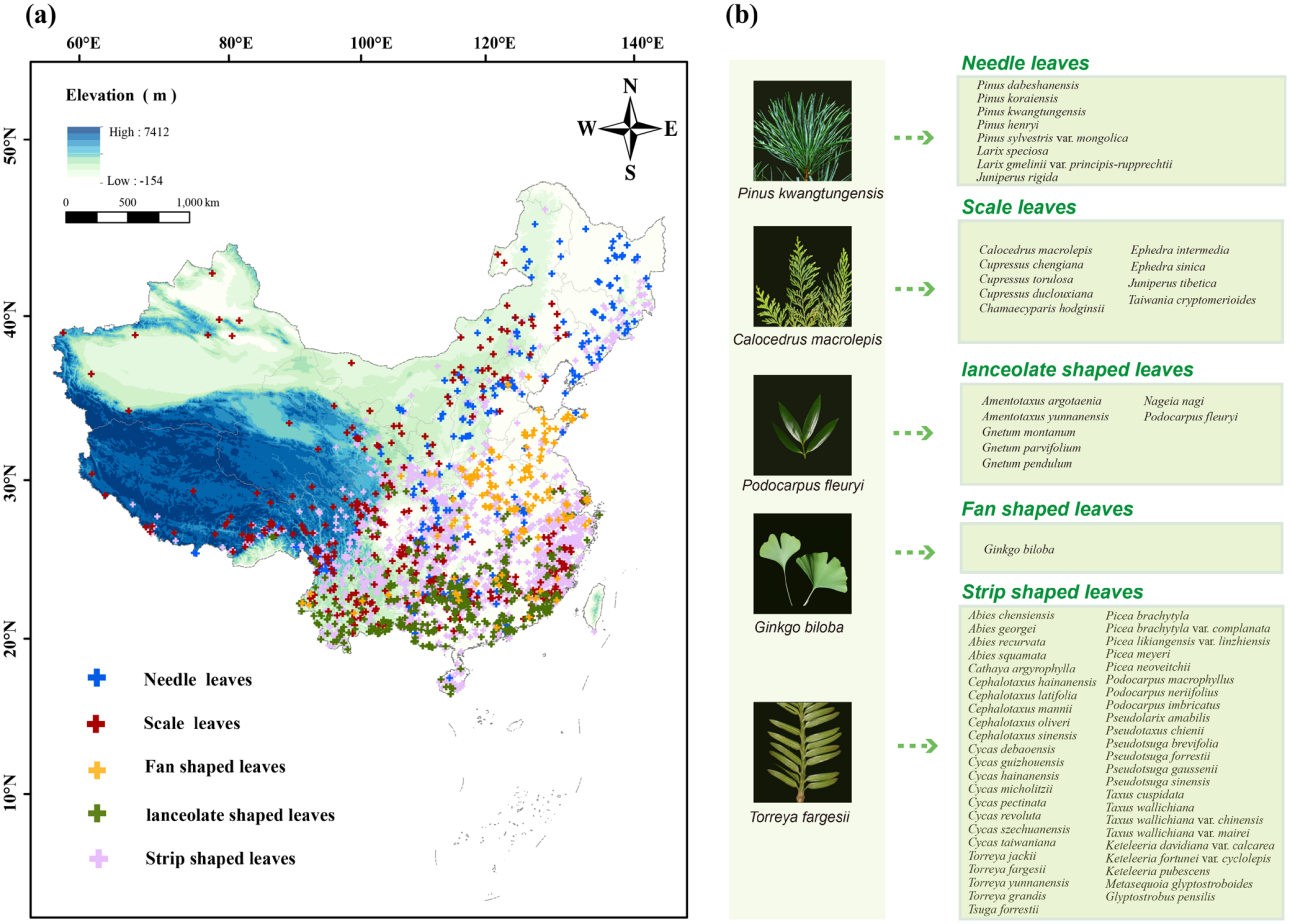
Geographic distribution and classifications of 71 rare gymnosperms (including varieties) in China. (a) Study area (China) and the spatial distribution of occurrence records of needle‐leaved gymnosperms (NLs), scale‐leaved gymnosperms (SLs), lanceolate‐shaped‐leaved gymnosperms (LSLs), fan‐shaped‐leaved gymnosperms (FSLs), and strip‐shaped‐leaved gymnosperms (SSLs). (b) The species list of different gymnosperm leaf shapes and the pictures of different leaf‐shaped gymnosperms.

The list of gymnosperm species in China was compiled from multiple authoritative sources: (1) the national list of key wild plants for protection (https://www.gov.cn/zhengce/2021–09/07/content_5727413.htm); (2) the Information System of Chinese Rare and Endangered Plants (ISCREP, https://www.iplant.cn/rep/protlist), which includes resources such as the *China Rare and Endangered Plants Illustrated Catalog*, *Red List of China's Biodiversity (Volume of Higher Plants)*, *Red Book of China's Plants*, and *Information on Threatened Species*; and (3) additional literature sources. Then the exact geographic distribution of these species was collected and organized through various approaches (Figure 1).

Species distribution data were obtained from the following sources: (1) fieldwork; (2) the National Plant Specimen Resource Centre (NPSRC, http://www.cvh.ac.cn/); (3) the National Specimen Information Infrastructure (NSII, http://www.nsii.org.cn/); (4) the Global Biodiversity Information Facility (GBIF, https://www.gbif.org/); and (5) published literature containing gymnosperm distribution points (Appendix S1). Duplicate records, records lacking coordinates, and erroneous data were removed. The'dism' package in R v.4.1.3 was used to filter occurrence points by thinning excessively dense data, randomly retaining only one record per 0.1° grid cell (Hijmans et al. 2011). After quality control, only species recorded at a minimum of 25 distinct locations were included in the modeling process. The final dataset was comprised of 71 species across 27 genera and 9 families, totaling 3825 distribution points (Appendix S1).

Morphological data on gymnosperm leaves were primarily collected from the *Flora of China* (https://www.iplant.cn/foc) and supplemented by additional literature. The 71 gymnosperm species (including varieties) were classified into five leaf morphology categories: needle leaves (NLs, 8 species), scale leaves (SLs, 9 species), lanceolate‐shaped leaves (LSLs, 7 species), fan‐shaped leaves (FSLs, 1 species), and strip‐shaped leaves (SSLs, 46 species).

### 2.2 Environmental Variables

Nineteen bioclimatic variables representing current climatic conditions (averages from 1970 to 2000) were obtained from the WorldClim 2.1 database (Fick and Hijmans 2017) at 2.5 arc‐minute (~5 km) resolution. Future climatic projections for the 2070s (average for 2061–2080) and the 2090s (average for 2081–2100) were downscaled from the MIROC6 global climate model (Kataoka et al. 2020) and based on Shared Socioeconomic Pathways (SSP) from the Coupled Model Intercomparison Project Phase 6 (CMIP6) (http://worldclim.org). Two CO_2_ emission scenarios, SSP2–4.5 representing medium emissions and SSP5–8.5 representing high emissions, were selected to simulate possible future climate conditions (Table 1).

**TABLE 1:** Environmental variables used in species distribution models.

Type	Code	Determinant environmental variables	Time period	Source
Bioclimatic variables	Bio1	Annual mean temperature (°C)	1970–2000	https://www.worldclim.org/
	Bio2	Mean diurnal range (°C)		
	Bio7	Temperature annual range (°C)		
	Bio15	Precipitation seasonality		
	Bio18	Precipitation of warmest quarter (mm)		
	Bio19	Precipitation of coldest quarter (mm)		
Environmental energy	AI	Aridity index		https://cgiarcsi.community/
	SRAD	Solar radiation (W/m^2^)	1983–2017	https://data.tpdc.ac.cn/
Topographical variables	EL	Elevation (m)	2019	https://www.resdc.cn/
	SLO	Slope		
	ASP	Aspect (°)		
Habitat variables	NPP	Net primary productivity	2019	http://www.nesdc.org.cn/
Soil condition	AWC	Available water capacity (mm/m)	2009	https://www.fao.org
	TT	Topsoil texture		
Human influence	HFI	Human footprint index	1995–2004	http://sedac.ciesin.columbia.edu/

To mitigate multicollinearity among the climate variables, Pearson correlation coefficients were calculated. For each pair of variables with a correlation coefficient greater than |0.8|, the less ecologically significant variable was excluded using the ‘Hmisc’ package in R (Harrell and Harrell 2019). This filtering process resulted in the retention of six key climatic variables: annual mean temperature (Bio1), mean diurnal range (Bio2), temperature annual range (Bio7), precipitation seasonality (Bio15), precipitation of warmest quarter (Bio18), and precipitation of coldest quarter (Bio19) (Figure S1a; Table 1).

In addition to the bioclimatic data, other environmental variables were incorporated to evaluate broader influences on gymnosperm distribution. Climatic variables under current conditions were combined with non‐climatic factors to assess the effects of topography, soil properties, and anthropogenic activities. After excluding variables with |*r*| ≥ 0.8, nine additional variables were selected, including aridity index (AI, 30 arc‐second), solar radiation (SRAD, 5 arc‐minute), elevation (EL, 3 arc‐second), slope (SLO, 3 arc‐second), aspect (ASP, 3 arc‐second), net primary productivity (NPP, 5 arc‐minute), available water capacity (AWC, 30 arc‐second), topsoil texture (TT, 30 arc‐second), and the human footprint index (HFI, 5 arc‐minute) (Figure S1b; Table 1). To ensure consistency across datasets, all environmental variables were standardized to a 2.5 arc‐minute resolution.

### 2.3 Modeling and Evaluation

This study developed ensemble species distribution models (eSDMs) through a consensus framework integrating seven complementary ecological niche modeling algorithms: classification tree analysis (CTA), flexible discriminant analysis (FDA), generalized boosted regression model (GBM), generalized linear model (GLM), maximum entropy (MAXENT), random forests (RF), and surface range envelope (SRE). This multi‐algorithm integration effectively mitigates methodological limitations inherent in single‐model approaches. All models were implemented using the default settings of the biomod2 package in R (Thuiller et al. 2009). To improve the accuracy of SDMs, 1000 pseudo‐absence coordinates were randomly generated within the distribution range of each gymnosperm species. These pseudo‐absence points were combined with occurrence data, and 75% of the combined dataset was randomly selected for model calibration, while the remaining 25% was used to evaluate model performance (Gong et al. 2020). The modeling process was repeated 10 times for each dataset to mitigate bias introduced by data partitioning and to improve the robustness and reliability of the results. Basal models with TSS (true skill statistic) values exceeding 0.75 were incorporated into the eSDMs using the committee averaging algorithm to ensure optimal predictive performance for occurrence probability (Zhang et al. 2020).

Model performance was assessed using two evaluation metrics: the area under the receiver operating characteristic curve (AUC) and the true skill statistic (TSS). AUC values range from 0 to 1, with values above 0.8 indicating high predictive accuracy (Xu et al. 2015). The TSS ranges from −1 to 1, with values closer to 1 reflecting greater model accuracy (Jiménez‐Valverde and Lobo 2007). The resulting eSDMs were applied to predict habitat suitability under present and future climate conditions (Table S1).

### 2.4 Changes in Species Range Change, Richness, and Centroid Migration

**FIGURE 2:**
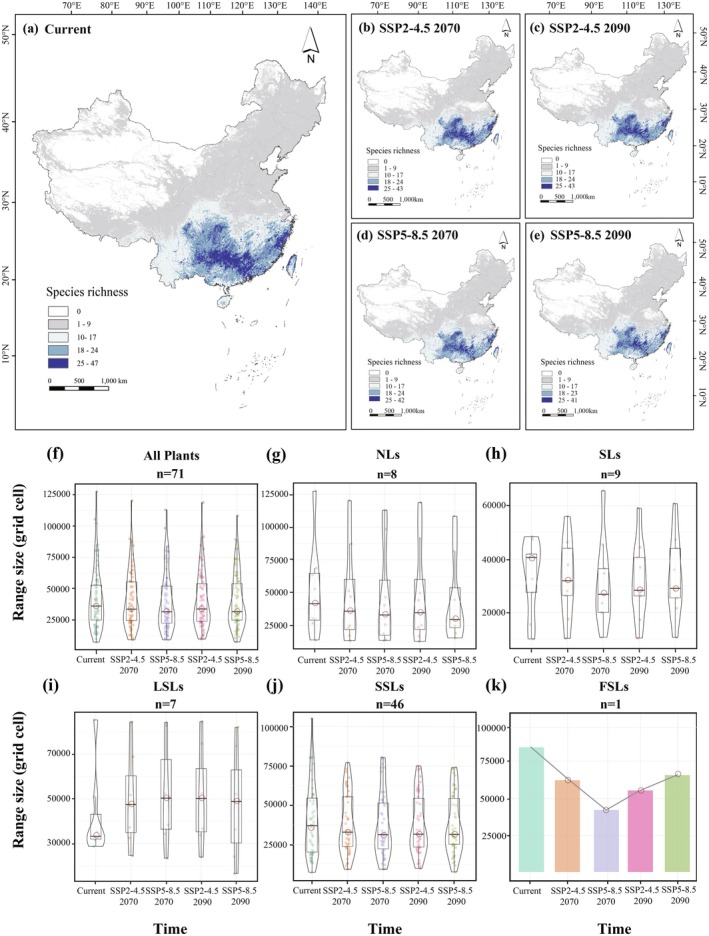
Changes in species richness distribution of gymnosperms in China from the current period to the 2090s. (a–e) Species richness maps for each period. (f–k) Range size change trends for all plants and needle‐leaved gymnosperms (NLs), scale‐leaved gymnosperms (SLs), lanceolate‐shaped‐leaved gymnosperms (LSLs), fan‐shaped‐leaved gymnosperms (FSLs), and strip‐shaped‐leaved gymnosperms (SSLs) from the current period to the 2090s.

Projection results for each species across the different time periods were aggregated using a weighted average method based on the TSS and AUC values of the calibrated models (Marmion et al. 2009). The ensemble projection outputs were subsequently converted into binary presence‐absence maps by applying a habitat suitability index threshold of 0.5. These maps were overlaid and analyzed using ArcGIS 10.8 (https://www.esri.com/). The SDMtoolbox was used to calculate area changes over time and generate species richness maps for each period. Visualization of species richness and distribution dynamics was performed using the ‘vioplot’ and ‘ggplot2’ packages in R (Figure 2; Figure S3).

**FIGURE 3:**
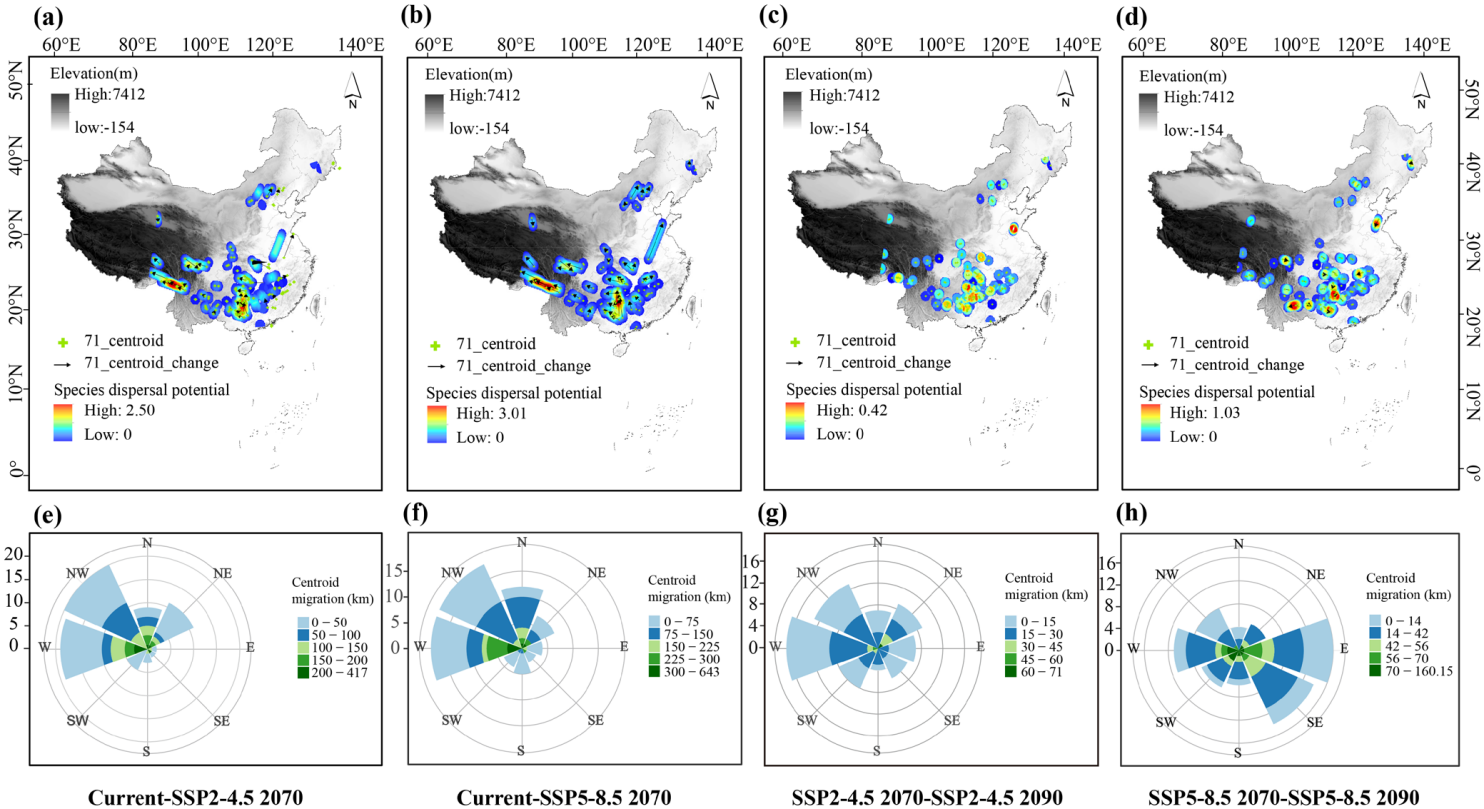
Centroid migration changes and potential dispersal corridors of gymnosperms in China from the current period to the 2090s. (a–d) Distance (magnitude) and direction of change at the centroid of the climatic niche (represented by the arrows) over each period. (e–h) The wind roses summarize the distance and direction of shift for 71 gymnosperms during each period. The direction of the middle sector is the migration direction, and the size of the sector represents the number of gymnosperm species migrating in this direction. The proportion of different colors that make up the fan represents the number of species, with the migration distance represented by that color.

To evaluate the geographic and spatial migration of gymnosperms across different climate periods, a multidimensional analysis incorporating longitude, latitude, and elevation was conducted. Centroid positions and potential dispersal corridors within suitable distribution areas were computed using the SDMtoolbox in ArcGIS 10.8. The latitude, longitude, and elevation of the centroids were extracted to quantify change amplitudes, decadal migration velocities, and directional shifts for each period (Figure 3; Figure S6; Table S3). Calculations followed established methodologies from prior studies (Liang et al. 2018).

### 2.5 Environment Factor and Climate Fluctuation Analysis

We utilized the environmental principal component analysis (PCAenv) method to characterize climate fluctuations experienced by gymnosperms and assess the environmental heterogeneity of the study area using R v4.2.3 (https://www.r‐project.org/) following the approach described by Jiang et al. (2018).

The principal components estimated from the analysis summarized the overall variation in six climate variables across populations during both present and future periods. Representative distribution points were selected using the “raster” package in R. To quantify environmental changes experienced by populations over time, we calculated the absolute difference between standardized PC1 scores from the future period and those from the present. After standardization, values closer to 0 indicate relative environmental stability, whereas values nearer to 1 indicate significant environmental changes. Environmental conditions for both the present and future periods, as well as the degree of environmental change since the present, were visualized using ArcGIS 10.8.

### 2.6 Identifying the Risk of Species Extinction

This study assessed changes in the distribution ranges of 71 rare gymnosperm species under future climate change scenarios by applying two extreme dispersal hypotheses. The first, the no dispersal assumption (ND), constrained projected future distributions within the boundaries of current climatic conditions. The second, the unlimited dispersal assumption (UD), allowed all modeled suitable areas to be considered as potential future distributions. Species range change was calculated as the percentage difference between climatic niche gain and loss. Changes in species threat levels were evaluated according to the IUCN Red List criterion A3 (IUCN 2024b). National nature reserve data were obtained from the publicly available records released by the Ministry of Ecology and Environment of China (https://www.mee.gov.cn/).

## 3 Result

### 3.1 Model Selection and Accuracy Evaluation

The evaluation of the eSDMs reveals that the mean TSS value across six models (CTA, FDA, GBM, GLM, MAXENT, and RF) exceeded 0.85, while the mean AUC value surpassed 0.90. In contrast, the SRE model produced average TSS and AUC values below 0.70, failing to meet the minimum evaluation criteria. As a result, the SRE model was assigned a lower weight in the construction of the eSDMs (Figure S2). Overall, the ensemble model exhibited high accuracy and reproducibility, indicating the reliability of the final predictions.

### 3.2 Effect of Environmental Factors on Gymnosperms

At the overall level, the analysis of environmental variable contributions indicated that Bio7, Bio1, and Bio19 were the top three factors, accounting for 35.8%, 23.6%, and 20.7% of the total contribution, respectively. Among non‐climatic variables, EL, AI, and HFI contributed 16.0%, 16.4%, and 9.0%, respectively. At the classification level, the relative importance of these three climatic variables exhibited slight variations, with Bio18 exerting a greater influence than Bio19 with NLs and FSLs. In addition, EL, AI, and HFI played a crucial role in determining suitable habitats for gymnosperms with diverse leaf morphologies (Figure S4; Table S4).

### 3.3 Future Spatiotemporal Shifts in Species Distribution and Richness

The results of the eSDMs showed significant changes in gymnosperm species richness and range size from the present to the 2070s and from the 2070s to the 2090s, reflecting a gradual declining trend (Figure 2a–e). At the overall level, more than 50% of gymnosperm species are projected to experience range reductions by the 2090s across both time periods (Figure 2f). At the classification level, from the present to the 2070s, a consistent reduction in range size was observed among species with NLs, SSLs, and FSLs. Notably, 81% of NLs, 56% of SLs, and 48% of SSLs species are expected to undergo habitat contraction, while FSLs ( *Ginkgo biloba* ) are projected to experience a nearly 17% reduction in their geographical range. In contrast, LSLs exhibited expansion trends, with 64% of species, respectively, increasing their distribution range. Between the 2070s and 2090s, the distribution patterns of all gymnosperm groups remained similar to those observed in the previous period, showing only minor fluctuations (Figure 2g–k; Table S2).

In the current period, the species richness hotspots are primarily concentrated in the eastern YunGui Plateau, the Nanling Mountains, and the Southeast Hills. By the 2070s and 2090s, the dynamics of species richness hotspots remained similar, although they exhibit a contraction relative to contemporary distributions and an expansion toward the southeastern part of the Sichuan Basin. Hotspot areas exhibited greater shrinkage and fragmentation under the SSP5‐8.5 scenario compared to SSP2‐4.5 (Figure 2a–e). At the classification level, the NLs are currently and primarily distributed in the eastern Inner Mongolia Plateau, the Changbai Mountains, and the eastern Sichuan Basin. Future projections indicate significant range contraction in the Inner Mongolia Plateau and Sichuan Basin, but northwestward expansion in the Changbai Mountains. SLs are largely concentrated in the southwestern mountainous regions, including the YunGui Plateau and the Hengduan Mountains. The species richness ranges of SLs will expand in the Hengduan Mountains and the southern Himalayas but contract markedly in the Wuyi Mountains, the Nanling Mountains, and the Sichuan Basin in the future. LSLs are predominantly distributed in southeastern coastal regions, such as the South China Hills. Under future climate scenarios, their high‐richness areas are projected to expand significantly northward. SSLs exhibit species richness distributions and future trends that are consistent with all gymnosperms, while FSLs are restricted to provinces in eastern and southern China. By the end of this century, suitable habitat ranges of FSLs are projected to contract significantly in the North China Plain and the middle and lower reaches of the Yangtze River (Figure S3).

### 3.4 Future Temporal Dynamics of Species Centroid Shifts

At the geographical scale, from the present period to the 2090s, gymnosperm migration hotspots are projected to concentrate in the western Hengduan Mountains, the southern Himalayas, and the western Nanling Mountains (Figure 3a–d). A northwestward shift is expected for the majority of gymnosperms between the present and the 2070s, with 57.7% (41 out of 71 species) under SSP2‐4.5 and 52.1% (37 out of 71 species) under SSP5‐8.5 following this trend (Figure 3g,h). At the spatial scale, about 55% of gymnosperms are expected to migrate to higher altitudes between the present and the 2070s, with this trend continuing from the 2070s to the 2090s. Four gymnosperm groups exhibit an initial migration to higher elevations, followed by a slight shift to lower altitudes. In contrast, the NLs exhibit the opposite trend, initially migrating to lower altitudes before shifting to higher altitudes under the SSP5‐8.5 scenario (Figure S6; Table S2).

From the present period to the 2070s, the projected migration distance for all gymnosperm species ranges from 82.62 km (SSP2‐4.5) to 120.43 km (SSP5‐8.5). Between the 2070s and the 2090s, the migration distance decreases, ranging from 16.27 km (SSP2‐4.5) to 36.59 km (SSP5‐8.5), indicating a shorter migration distance compared to the preceding period. Specifically, shifts in elevation amplitude and centroid displacement of potential distribution areas gradually diminish from the present to the 2090s, while the rate of change per decade exhibits an increasing trend. Across all time periods, the centroid migration distance and decadal rate of change are consistently higher under the high‐emission scenario (SSP5‐8.5) than under the low‐emission scenario (SSP2‐4.5) (Table S3). Gymnosperms under SSP5‐8.5 exhibit greater centroid displacement and higher 10‐year change velocities compared to SSP2‐4.5 across all four time periods.

### 3.5 Environmental Heterogeneity and Climate Variability

**FIGURE 4:**
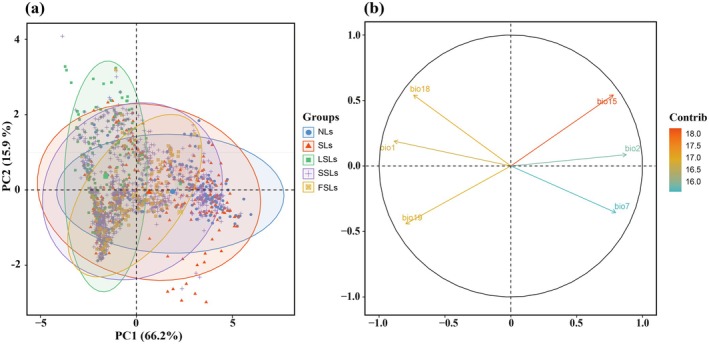
PCA analysis of six bioclimatic variables of five leaf‐shaped gymnosperms (a, b). Needle‐leaved gymnosperms (NLs), scale‐leaved gymnosperms (SLs), fan‐shaped‐leaved gymnosperms (FSLs), lanceolate‐shaped‐leaved gymnosperms (LSLs), strip‐shaped‐leaved gymnosperms (SSLs).

The PCA results revealed significant differences in numerical ranges of environmental factors among LSLs, NLs, and the distinct groups of SLs, SSLs, and FSLs, indicating notable ecological divergence across these groups. The Bio15 emerged as the primary climatic factor driving significant divergence in the environmental niches of different plant groups (Figure 4).

**FIGURE 5:**
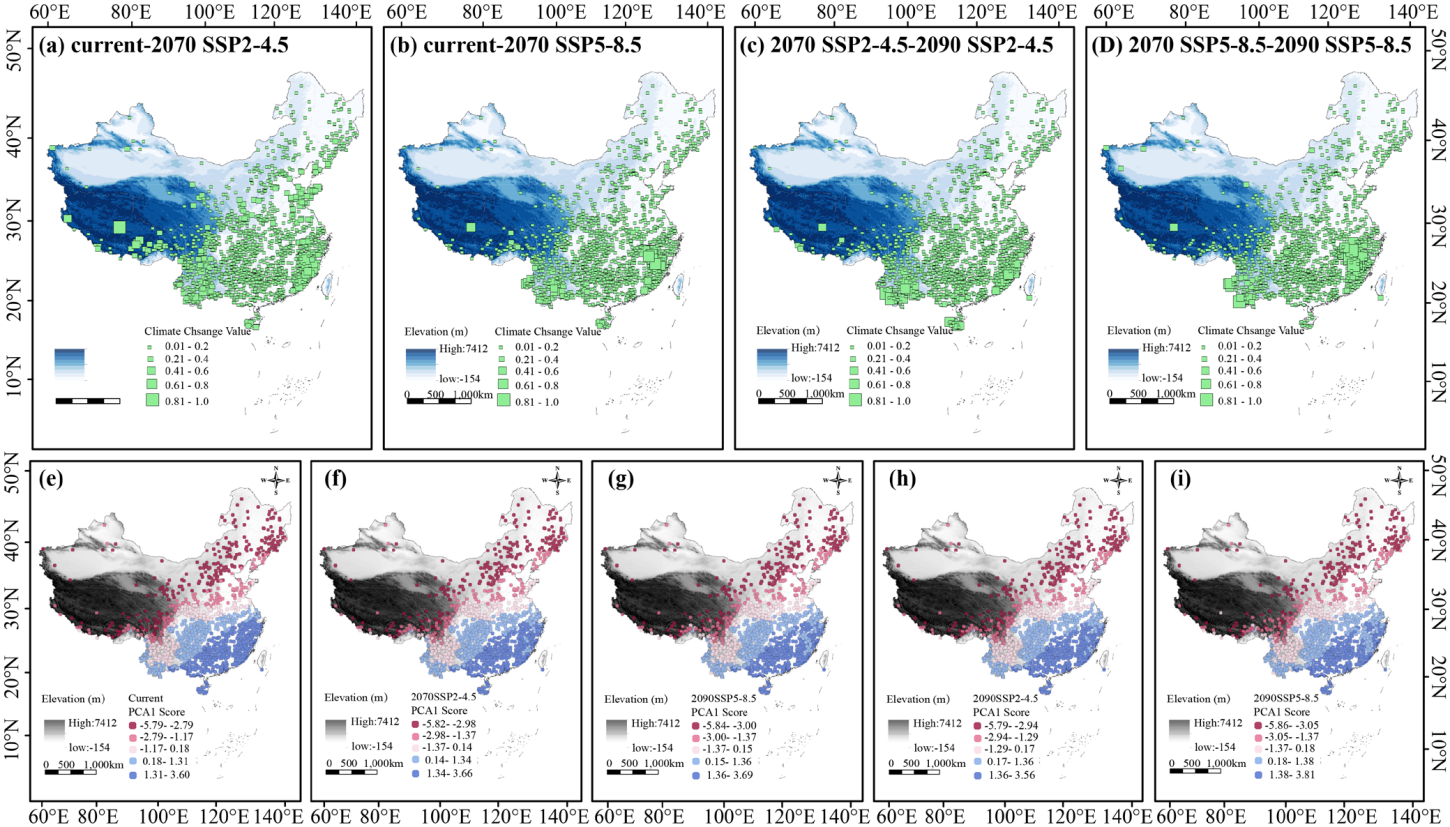
Environmental heterogeneity in the study area and climate fluctuations for rare gymnosperms in China from the current period to 2090. (a–d) Climate fluctuations of the representative distribution points for gymnosperms from current to 2090. The sizes of green dots represent the change in six bioclimatic variables from the current period to 2090. (e–i) Environmental heterogeneity in the study area based on 15 environmental variables during the current period. Dots represent current period PCA1 scores of the study area, and dot colors represent the different ranges of PCA1 scores estimated from six bioclimatic variables.

Analysis of environmental heterogeneity across four time periods revealed a stepwise variation in environmental conditions, transitioning from southeastern China—including the Southeast Hills and Yungui Plateau—toward the northwestern regions, such as the Loess Plateau and Qinghai‐Tibet Plateau. This shift corresponds to the extension of the monsoon belt and increasing latitude. As time progresses and climate fluctuations intensify, the environmental heterogeneity of southeastern coastal regions, such as the Zhejiang‐Fujian Hills, is projected to increasingly resemble the environmental conditions of the North China Plain and the mid‐ and lower reaches of the Yangtze River Plain. This trend is expected to be more pronounced by the 2090s (Figure 5e–i). Climatic fluctuation analysis indicates significant variability in the southern Himalayas, southwestern Hengduan Mountains, and southeastern Zhejiang‐Fujian Hills from the present to the 2070s (Figure 5a,b). From the 2070s to the 2090s, climate fluctuations are projected to intensify further in the Zhejiang‐Fujian Hills and southwestern Yunnan‐Guizhou Plateau, while the southern Himalayas are anticipated to experience a reduction in climate variability (Figure 5c,d).

### 3.6 Identifying Conservation Gaps for Gymnosperm Species

Across both extreme dispersal hypotheses, the number of threatened gymnosperm species is higher in the high‐emission scenario (SSP5‐8.5) compared to the medium‐emission scenario (SSP2‐4.5), while fewer species are classified as least concern. *Abies recurvata* consistently experiences a distribution area reduction exceeding 80% across all hypotheses and climate scenarios, resulting in its classification as critically endangered. Under ND nearly one‐third of gymnosperms are projected to lose 10% of their suitable habitat from the present to the 2070s (25 out of 71 species under SSP2‐4.5; 26 out of 71 species under SSP5‐8.5). This trend continues from the present to the 2090s, with 27 species affected under SSP2‐4.5 and 29 species under SSP5‐8.5. Specifically, between the present and the 2070s, eight species under SSP2‐4.5 and six species under SSP5‐8.5 are expected to experience such habitat loss. By the 2090s, this number shifts to six species under SSP2‐4.5 and 11 species under SSP5‐8.5 (Table S5).

**FIGURE 6:**
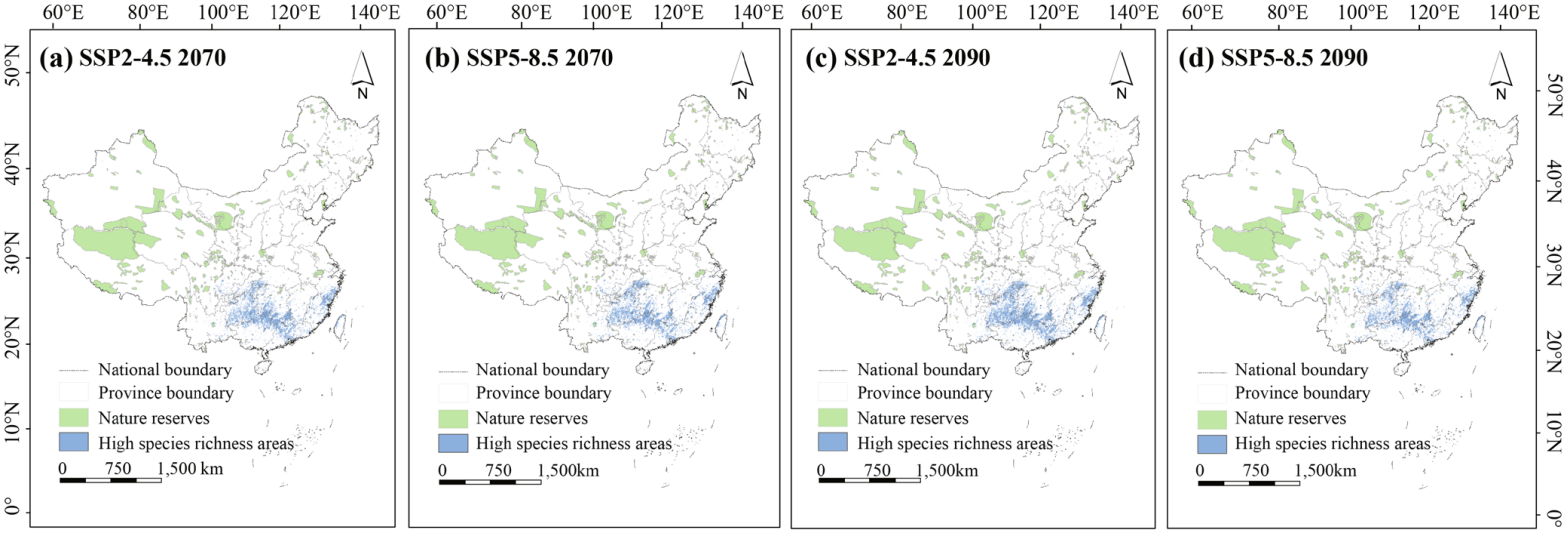
High species richness areas of gymnosperms in China from the 2070s to 2090s in each scenario. The green areas represent the National Nature Reserve, and the blue areas represent the high species richness areas.

Under UD many species are projected to gain new potential suitable habitats. However, more than half of the species are still expected to experience habitat loss. From the present to the 2070s, 36 out of 71 species under SSP2‐4.5 and 39 out of 71 species under SSP5‐8.5 are predicted to lose part of their habitat. This trend continues from the present to the 2090s, with 36 species affected under SSP2‐4.5 and 35 species under SSP5‐8.5. Under the high‐emission climate change scenario (SSP5‐8.5), *Cathaya argyrophylla*, *Ephedra sinica* , *Ginkgo biloba* , *Metasequoia glyptostroboides* , *Torreya grandis*, *Taiwania cryptomerioides*, *Pinus dabeshanensis*, and *Picea meyeri* are predicted to lose over 50% of their habitat, placing them at exceptionally high risk of extinction (Table S6). In addition, gymnosperm species richness hotspots, such as the Nanling Mountains, the eastern Sichuan Basin, and the East China Mountains, exhibit limited overlap with national nature reserves across all four time periods (Figure 6).

## 4 Discussion

### 4.1 Environmental Variables Affecting the Distribution of Gymnosperms

Climate, topography, soil, and anthropogenic factors significantly influence plant growth and development, particularly for relict gymnosperms with limited distribution ranges, fragmented habitats, and sparse wild populations (Tagliari et al. 2021). This study identifies Bio7, Bio1, and Bio19 as the three pivotal climatic variables shaping the distribution of gymnosperms within their ecological niches. In China, the MAT ranges from −25°C to 25°C, while the temperature range spans from 11°C to 59.7°C. Higher latitudes in northern China typically experience lower temperature extremes and greater temperature variability compared to the southern regions (Pandey et al. 2020). These temperature extremes likely correlate to the restricted distribution of gymnosperms in northern China, with the majority occurring south of the Qinling Mountains. Climatic fluctuations during the Late Tertiary and Quaternary glaciations further contributed to temperature instability, disrupting the growth and developmental cycles of gymnosperms. This disruption extended generation times, reduced reproductive capacity, and led to range contractions for species such as *Abies ziyuanensis* and *Thuja sutchuenensis* (Yao et al. 2021). Beyond stable temperature, sufficient winter–spring precipitation is critical for endemic alpine plants in China's Hengduan Mountains and the eastern Yunnan‐Guizhou Plateau. Its consistent precipitation prevents seed dehydration during winter and promotes germination and branch growth in spring, sustaining regional diversity (Huang et al. 2015; Zheng et al. 2017). As a result, these regions have become the biodiversity hotspot of gymnosperms, harboring relict, endangered, and endemic taxa such as *Davidia involucrata* and *Metasequoia glyptostroboides* , and function as critical refuges for future adaptation (Lü et al. 2018; Tang et al. 2018).

Climatic factors play a crucial role in shaping the geographical distribution of plants with various leaf morphologies. The findings of this study indicate that Bio15 precipitation seasonality is the primary climatic factor driving spatial distribution differences among gymnosperms with various leaf shapes (Figure 4; Figure S3). Moisture availability is closely related to seed germination, seedling growth, and population regeneration, making species distribution heavily dependent on local precipitation levels, which can fluctuate over time. Temperate and alpine plants, once widespread in southwestern China, are experiencing contractions in their suitable ranges due to persistent water scarcity, resulting in migration toward eastern regions with higher precipitation levels (Lu et al. 2020). Gymnosperms with lanceolate or broader leaves are predominantly distributed in warm, precipitation‐rich subtropical and tropical monsoon rainforests at low to mid‐elevations in southern China. In contrast, needle‐shaped, scale‐shaped, and narrow‐leaved species frequently inhabit temperate monsoon coniferous, broad‐leaved mixed forests, and subtropical evergreen broad‐leaved forests, with lower seasonal precipitation, cooler temperatures, and higher latitudes and altitudes providing favorable conditions for species adapted to drier environments (Wang and Wang 2015; Wright et al. 2017; Li et al. 2021). Plants with larger leaves generally require greater water inputs and thrive in warmer, more humid environments. This is because the carbon gain from photosynthesis in large‐leaved plants is often outweighed by the water loss through transpiration, while the increased temperature difference between the leaf surface and the surrounding air can cause thermal damage (Givnish 1984; Okajima et al. 2012; Scoffoni et al. 2011). These factors underscore the critical influence of precipitation in determining the geographical distribution of plants with different leaf morphologies.

In this study, EI was identified as a key environmental factor influencing the geographic distribution patterns of gymnosperms (Figure S4). Compared to angiosperms, gymnosperms exhibit distinct morphological traits such as narrow, small leaves, low specific leaf area, and high wood density. This morphological plasticity allows gymnosperms to reduce transpiration efficiency, regulate internal temperature and water balance, and enhance photosynthetic capacity by decreasing leaf area or increasing leaf thickness at higher altitudes. These adaptive strategies improve frost tolerance and physiological function, enabling gymnosperms to survive low‐temperature, high‐altitude conditions (Caspersen et al. 2018; Lintunen et al. 2018). Transitional zones along altitudinal gradients create synergistic interactions between mountain environments and climatic factors, fostering significant habitat heterogeneity. This heterogeneity provides diverse habitat types and environmental resources for gymnosperms (Cun and Wang 2010; Wang et al. 2018). Additionally, reduced interspecific competition and lower anthropogenic disturbances at higher altitudes further enhance gymnosperm survivability (Tang et al. 2018; Perrigo et al. 2020). The HFI also emerged as a significant contributor to the model prediction results. In conclusion, the relationship between rare gymnosperms and environmental factors is complex, driven by the synergistic effects of multiple variables. These interactions collectively shape the geographical distribution and diversity of gymnosperms, underscoring the need for comprehensive conservation strategies.

### 4.2 Effects of Climate Change on Gymnosperms in China

Quaternary climate fluctuations and current environmental conditions have significantly shaped the geographic distribution patterns of gymnosperms, predominantly resulting in negative impacts (Umair et al. 2023). The results of this study indicate that climate change will continue to adversely affect gymnosperms in China, leading to reductions in overall population sizes and species richness. The current distribution of gymnosperms in China reveals higher species richness in the southern regions and lower richness in the north, with concentrations observed at mid‐ to low latitudes south of the Yellow River, particularly in the Yunnan‐Guizhou Plateau and along the southern ridge of Guangdong Province (Figures 1 and 2a). Many of these areas fall within subtropical and temperate monsoon zones, characterized by consistent annual sunlight duration, stable solar angles, minimal temperature fluctuations, and relatively arid and cool climates. Gymnosperms demonstrate strong adaptability to these environmental conditions (Li et al. 2021; Taheri et al. 2021). By the 2070s, the richness of gymnosperms is projected to increase significantly in the southern Tibetan Plateau, the southeastern Yunnan‐Guizhou Plateau, and the Hengduan Mountains compared to current conditions. Conversely, species richness is expected to decline in the middle and lower reaches of the Yangtze River Plain, the northern Sichuan Basin, and the southeastern hills. This trend is more pronounced under the SSP5‐8.5 scenario. Between the 2070s and the 2090s, regions that previously experienced a decline in species richness are anticipated to exhibit a slight increase in species abundance. However, compared to the present, the overall species richness in these areas will still exhibit a downward trend (Figure S5). Additionally, under future high CO_2_ emission scenarios, regions with high species richness are expected to shift toward the western Hengduan Mountains, the southern Himalayas, and the western Nanling Mountains. The southern Wuyi Mountains and southwestern Yunnan‐Guizhou Plateau are predicted to experience significant climatic variations in the coming decades (Figures 3 and 5). The migration of gymnosperms from these regions reflects their preference for environments with stable climatic conditions, facilitating additional opportunities for habitat establishment and persistence (Pandey et al. 2020).

Under future climate change scenarios, gymnosperms are expected to migrate predominantly toward high‐altitude mountainous regions in the northwest (Figure 3; Figure S6), aligning with previous findings (Iverson et al. 2008). As typical arboreal plants, gymnosperms exhibit significantly greater sensitivity to spring temperature fluctuations compared to shrubs and are less affected by chilling accumulation at higher elevation areas. In the context of global warming, higher elevations experience more rapid warming compared to lower elevations. This accelerates spring phenological phases, promotes xylem cell growth, and increases carbon assimilation rates, thereby providing gymnosperms with a competitive advantage at higher altitudes and driving their migration to these regions (Gao et al. 2022; Li et al. 2023). The expansion and migration of gymnosperms primarily occur through seed dispersal and seedling regeneration; however, the limited rate of dispersal significantly constrains their ability to expand distribution ranges (Iverson et al. 2004). With ongoing climate warming and increased humidity, the frequency of extreme climatic events has risen, exerting negative impacts on gymnosperm habitats. This combination of factors has synergistically contributed to the reduction of species range sizes and the worsening of the threatened status of gymnosperms.

The distribution patterns of the five gymnosperm leaf types exhibit distinct trends of change. From the present period to the 2090s, the ranges of LSLs are projected to expand significantly under both climate scenarios, driven by rising global temperatures. In contrast, the average range of other angiosperms is expected to contract. Plant leaf morphology has been shown to vary significantly in response to different environmental conditions. In cold and arid climates, deciduous tree species characterized by small and pointed leaves are prevalent. In contrast, in warm and humid climates, evergreen tree species with medium to large leaves and elliptical leaf shapes are frequently encountered (Li and Wang 2021; Li et al. 2020; Chen et al. 2025). Currently, most gymnosperms typically possess needle‐shaped, scale‐shaped, or strip‐shaped leaves, characterized by sunken stomata, a well‐developed cuticle, and a low specific leaf area. These traits enhance leaf resource utilization and confer resistance to various environmental stresses (Pittermann et al. 2012; Rueda et al. 2017). Compared to angiosperms, gymnosperms demonstrate slower initial growth and lower maximum growth efficiency and are predominantly restricted to the regions characterized by low angiosperm growth rates, such as dry or cold environments (Bond 1989). However, as global warming intensifies, certain gymnosperm taxa, including species within the Podocarpaceae and Taxaceae families, have begun to exhibit adaptive leaf traits, resulting in flatter and broader leaf morphologies, such as lanceolate forms (Biffin et al. 2012). These angiosperm‐like leaf structures improve photosynthetic efficiency and water transport, promoting growth and development in warmer, more humid environments and enhancing competitiveness with angiosperms (Lü et al. 2018). Overall, gymnosperm species, due to their unique life cycles and heightened sensitivity to climatic conditions, are undergoing evolutionary processes that may lead to the development of novel adaptive characteristics and the differentiation of new species in response to future climate change.

### 4.3 Assessing Gymnosperm Vulnerability Under Future Scenarios

According to the latest IUCN Red List classification and criteria, this study included a total of 38 threatened gymnosperm species, of which four were classified as Critically Endangered (CR), 10 as Endangered (EN), and 24 as Vulnerable (VU) (IUCN 2024a). The majority of the remaining species are also considered rare, relict, or nationally protected gymnosperms in China. Under two extreme dispersal scenarios, the habitat loss risk assessment indicates that 13 gymnosperm species are projected to experience significant contractions in their suitable habitats, leading to an increased threat status. These species include *Abies chensiensis*, *Abies recurvata*, *Cupressus torulosa* , *Ephedra sinica* , *Larix gmelinii* var. *principis‐rupprechtii*, *Gnetum pendulum*, *Pinus dabeshanensis*, *Pseudotsuga gaussenii*, *Picea meyeri*, *Pinus koraiensis* , *Taxus cuspidata* , *Torreya grandis*, and *Taiwania cryptomerioides* (Tables S5 and S6). Notably, some gymnosperms remain under threat and require ongoing monitoring, even if their risk of extinction has not escalated to a higher classification. Previous studies indicate that approximately 41% of China's 109 gymnosperm species are expected to face extinction risks and declining species richness due to climate change between 2000 and 2050 (Wu 2020). Furthermore, this study found that over half of the needle‐leaved gymnosperms and one‐third of the scale‐leaved gymnosperms exhibited a more pronounced increase in endangerment compared to other groups, accounting for six of the 13 species projected to face heightened risk. Conversely, among the species with increased endangerment levels, only one has lanceolate leaves. This trend may be attributed to the adaptation of these species to cold climates, which renders them less capable of coping with the elevated temperatures associated with global warming. As a result, small‐leaved gymnosperms may face a greater survival crisis in adapting to global warming (Agurla et al. 2018; Jin et al. 2023), highlighting the urgent need for immediate conservation measures and protective actions.

Currently, nature reserves in China account for approximately 14.9% of the nation's land area and cover 35.89% of the near‐threatened plant diversity hotspots, playing a crucial role in biodiversity conservation (MEP 2016). Despite this, prior research has demonstrated that the effectiveness of these reserves in achieving conservation objectives remains inadequate. Conservation gaps persist in these regions (Huang et al. 2016; Xia et al. 2022; Xue et al. 2023). Only 8.0% of high‐priority regions for gymnosperms fall within the boundaries of nature reserves (Li et al. 2021). Future climate change presents a significant threat to the habitats of multiple gymnosperms, potentially accelerating the decline of natural populations. Although rare gymnosperms may migrate northwestward, with new suitable habitats emerging near the Fanjingshan and Wuyi mountain ranges, a considerable reduction in the areas previously characterized by high species richness is predicted. This trend aligns with findings from previous research (Ye et al. 2021). Under current and future climate scenarios, nearly half of the gymnosperms (25–47 species) are distributed across the Nanling Mountains, the eastern Sichuan Basin, and the East China Mountains. However, the overlap between these regions of high species richness and existing national nature reserves remains limited (Figure 6), posing a significant challenge to gymnosperm conservation. To address these gaps, it is proposed that specific conservation and sustainable utilization measures be implemented. These may include expanding the boundaries of existing nature reserves to encompass critical habitats, establishing new protected areas aligned with projected species distributions, and enhancing artificial introduction and cultivation to support species migration and bolster conservation efforts (Fragnière et al. 2015; Xiao et al. 2024). Furthermore, regular monitoring of population dynamics and the health status of protected species is essential.

It is important to note that the predicted patterns of gymnosperm species richness may exhibit slight discrepancies from actual observed patterns. These differences can be attributed to variations in the number of species selected, inconsistencies in species distribution data, and the computational models employed (Ye et al. 2021). Such uncertainties add complexity to species conservation and management, necessitating field investigations and adaptive management strategies to effectively address species endangerment challenges. The potential distribution areas identified in this study offer valuable insights into gymnosperms' responses to environmental changes, serving as a critical foundation for the development of informed and effective conservation strategies. By enhancing understanding of distribution dynamics, these findings contribute to the establishment of sustainable practices aimed at preserving gymnosperm diversity and mitigating future biodiversity loss.

## 5 Conclusions

This study examined the responses of 71 rare gymnosperm species (including varieties) to future climate scenarios in China, focusing on shifts in their geographical distribution ranges. The analyses revealed that over half of the gymnosperm species are projected to experience consistent range contractions and shift northwestward between the present and 2090 as a result of climate warming. Additionally, numerous gymnosperms are expected to migrate from lower to higher elevations from the present to 2090. The Bio15 emerged as the primary factor driving divergence in the environmental niches of gymnosperms with different leaf morphologies. Although certain gymnosperm species are predicted to expand their ranges, the majority of habitats will remain susceptible to increased climate variability, exacerbating the vulnerability of these populations. Furthermore, the threat status of some gymnosperms is anticipated to deteriorate. Under future climate change scenarios, the eastern Yunnan–Guizhou Plateau, the Nanling Mountains, and the Southeast Hills are projected to become new hotspots for gymnosperm diversity. To ensure the effective and efficient conservation of gymnosperms, additional field studies are necessary to investigate the spatiotemporal dynamics of species in ecologically sensitive areas.

## Author Contributions

**Jinyi Fu:** conceptualization (equal), data curation (lead), formal analysis (lead), investigation (lead), methodology (lead), software (lead), visualization (lead), writing – original draft (lead), writing – review and editing (equal). **Wenjie Song:** data curation (equal), formal analysis (equal), investigation (equal), visualization (equal). **Chuncheng Wang:** formal analysis (equal), investigation (equal), visualization (equal). **Xiaolong Jiang:** methodology (equal), software (equal), visualization (equal). **Xiangbao Shen:** methodology (equal), software (equal). **Rong Yi:** conceptualization (lead), funding acquisition (supporting), investigation (equal), project administration (lead), resources (lead), supervision (lead), writing – original draft (equal), writing – review and editing (lead).

## Conflicts of Interest

The authors declare no conflicts of interest.

## Supporting information


**Figure S1:** ece371980‐sup‐0001‐FiguresS1‐S6.docx.

**Table S1:** ece371980‐sup‐0002‐TablesS1‐S6.docx.

**Appendix S1:** ece371980‐sup‐0003‐AppendixS1.xlsx.

## Data Availability

The data that supports the findings of this study are available in the Supporting Information of this article.
