# Supplementary material for: Spatiotemporal Distribution Patterns and Conservation Priorities of Gymnosperms With Different Leaf Shapes in China Under Climate Change
Source: Ecol Evol. 2025 Aug 14;15(8):e71980. doi: 10.1002/ece3.71980 (PMC12354979; doi:10.1002/ece3.71980)
Supplement: Supplementary file 1 — Figure S1: ece371980‐sup‐0001‐FiguresS1‐S6.docx. [file ECE3-15-e71980-s002.docx]

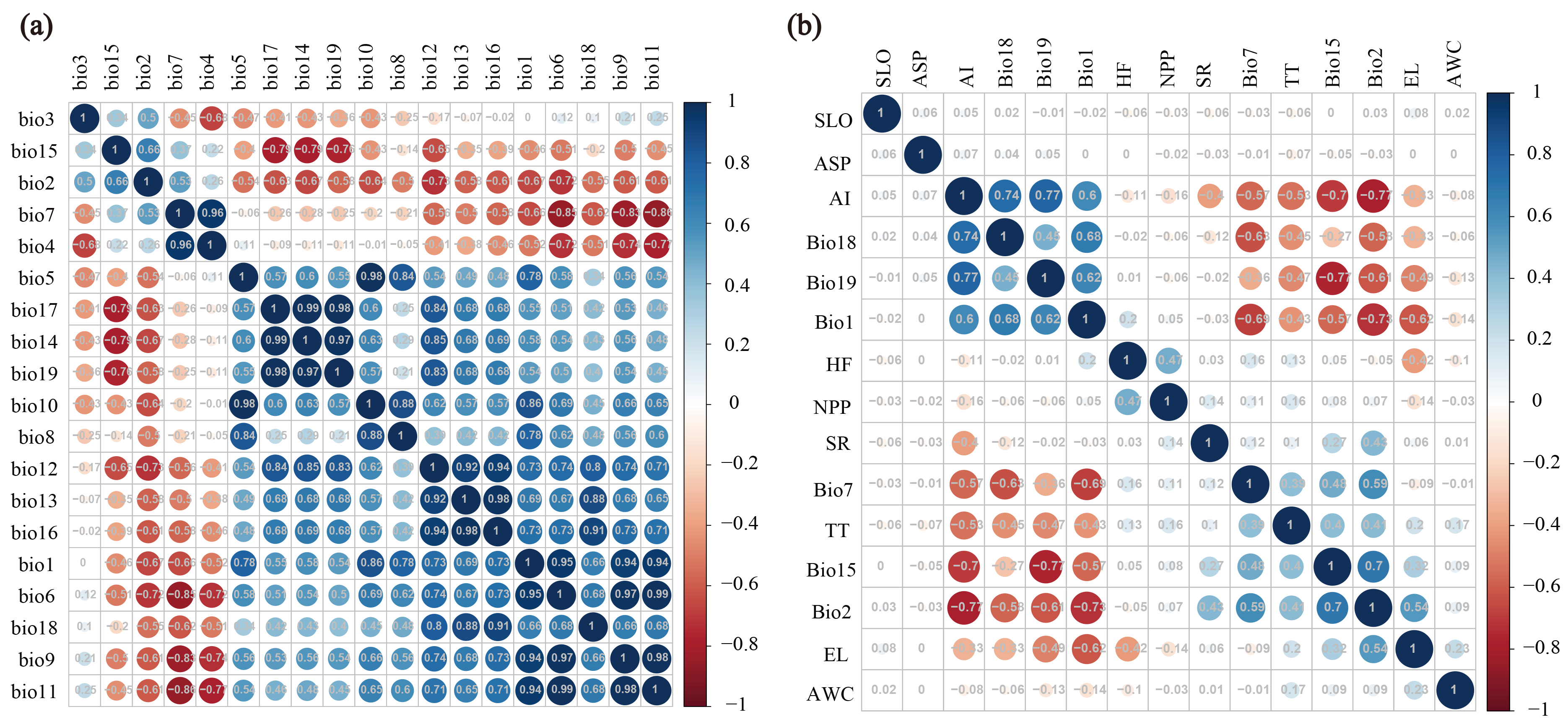


Figure. S1. Pearson correlation analysis and multicollinearity test for the climate (a) and all environmental variables (b). Bio1: Annual Mean Temperature; Bio2: mean diurnal range; Bio7: Temperature Annual Range; Bio15: precipitation seasonality; Bio18: Precipitation of Warmest Quarter; Bio19: Precipitation of Coldest Quarter; AI: aridity index; SR: solar radiation; AWC: available water capacity; EL: elevation; SLO: slope; ASP: aspect; NPP: net primary productivity; TT: topsoil texture; HFI: human footprint index.


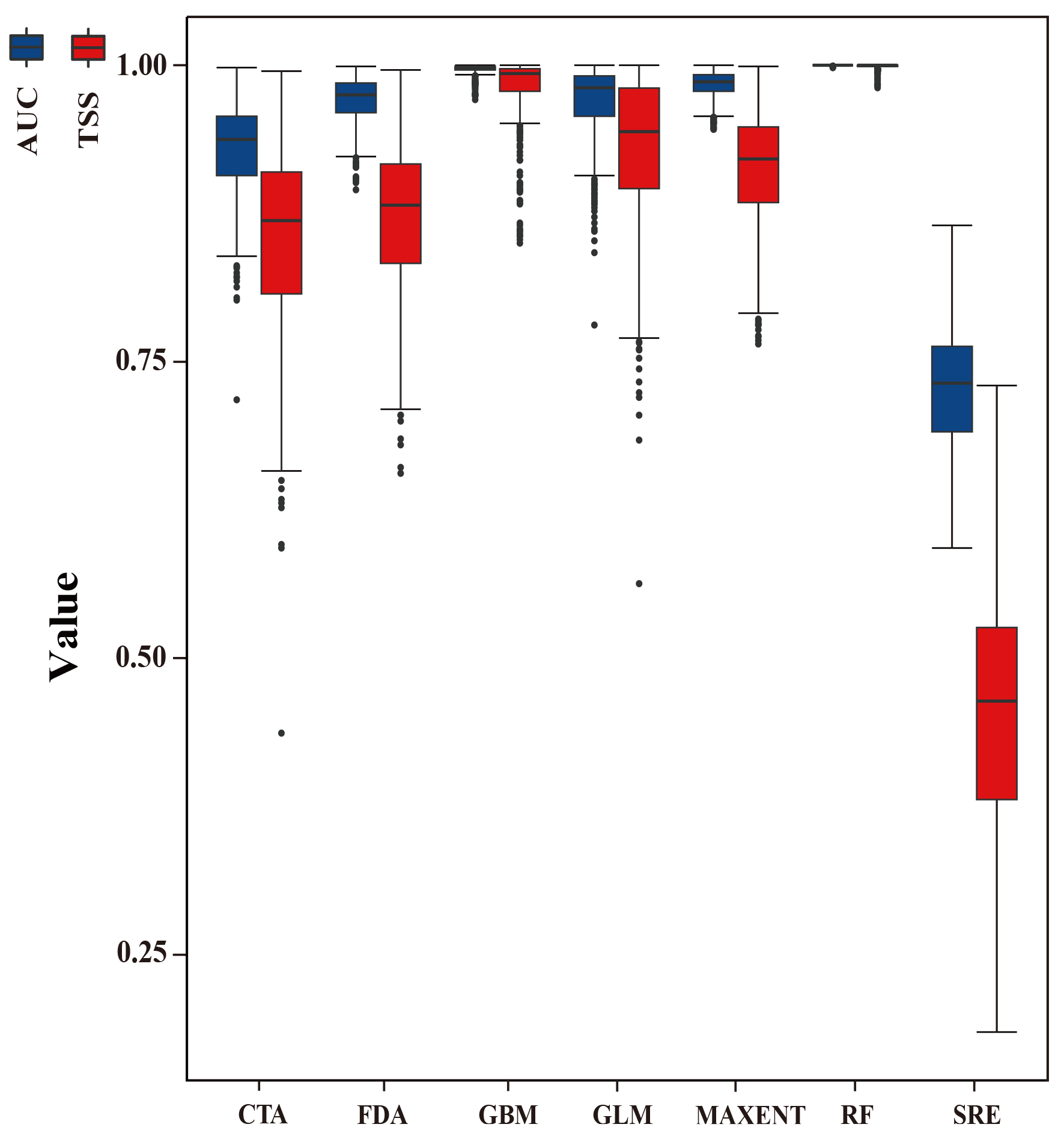


Figure. S2. Accuracy of prediction results from different models with the participation of fifteen environmental variables. CTA: classification tree analysis; FDA: flexible discriminant analysis; GBM: generalized boosted regression model; GLM: generalized linear model; MAXENT: maximum entropy; RF: random forests; SRE: surface range envelope.


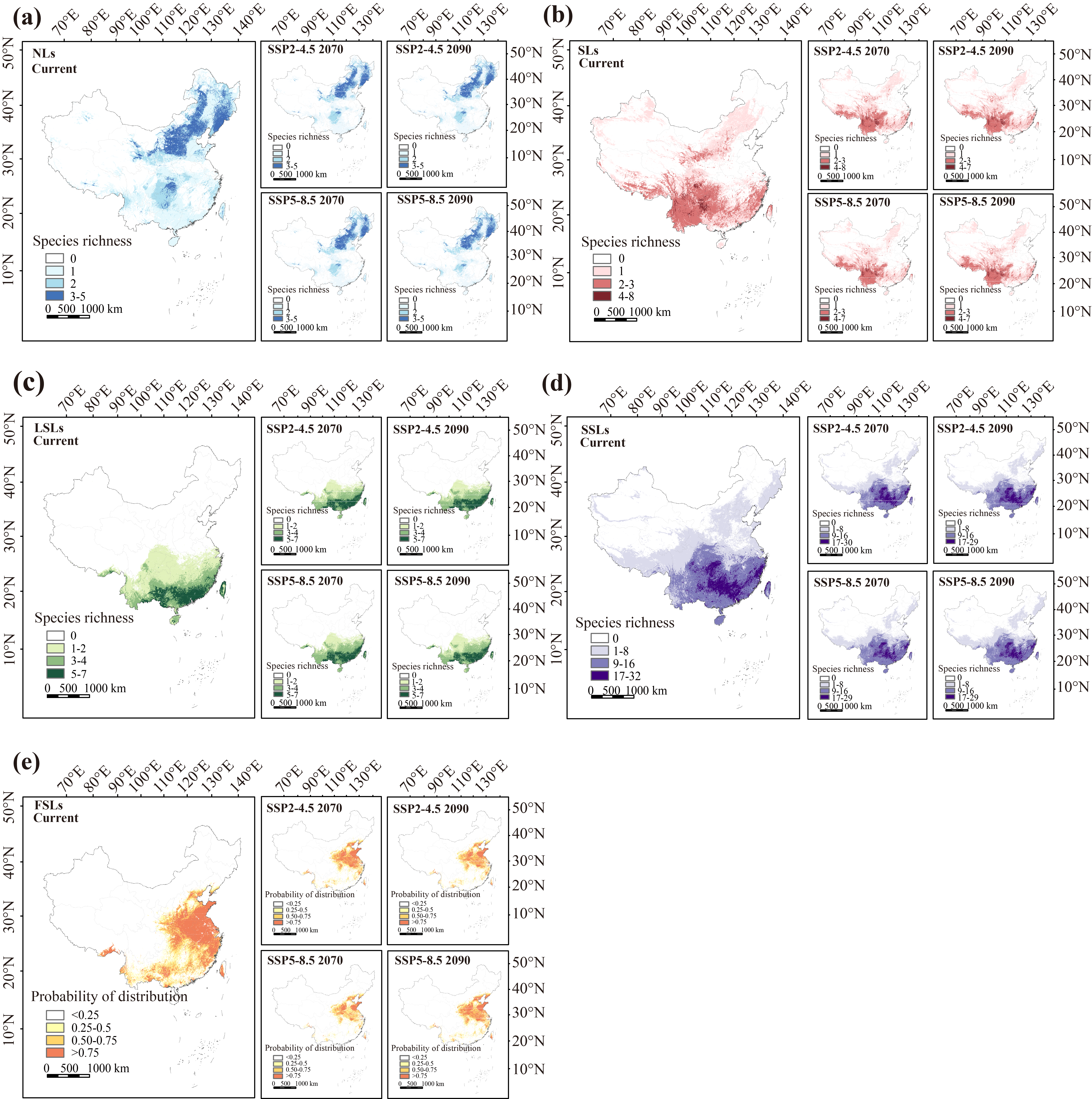

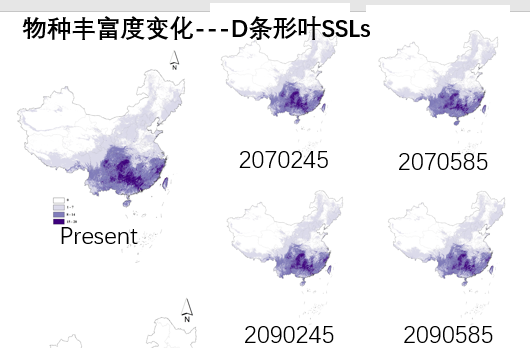


Figure. S3. Changes in species richness distribution of different leaf-shaped gymnosperms in China from the current period to 2090s. (a–e) needle leaves gymnosperms (NLs), scale leaves gymnosperms (SLs), lanceolate-shaped leaves gymnosperms (LSLs), fan-shaped leaves gymnosperms (FSLs), and strip-shaped leaves gymnosperms (SSLs).


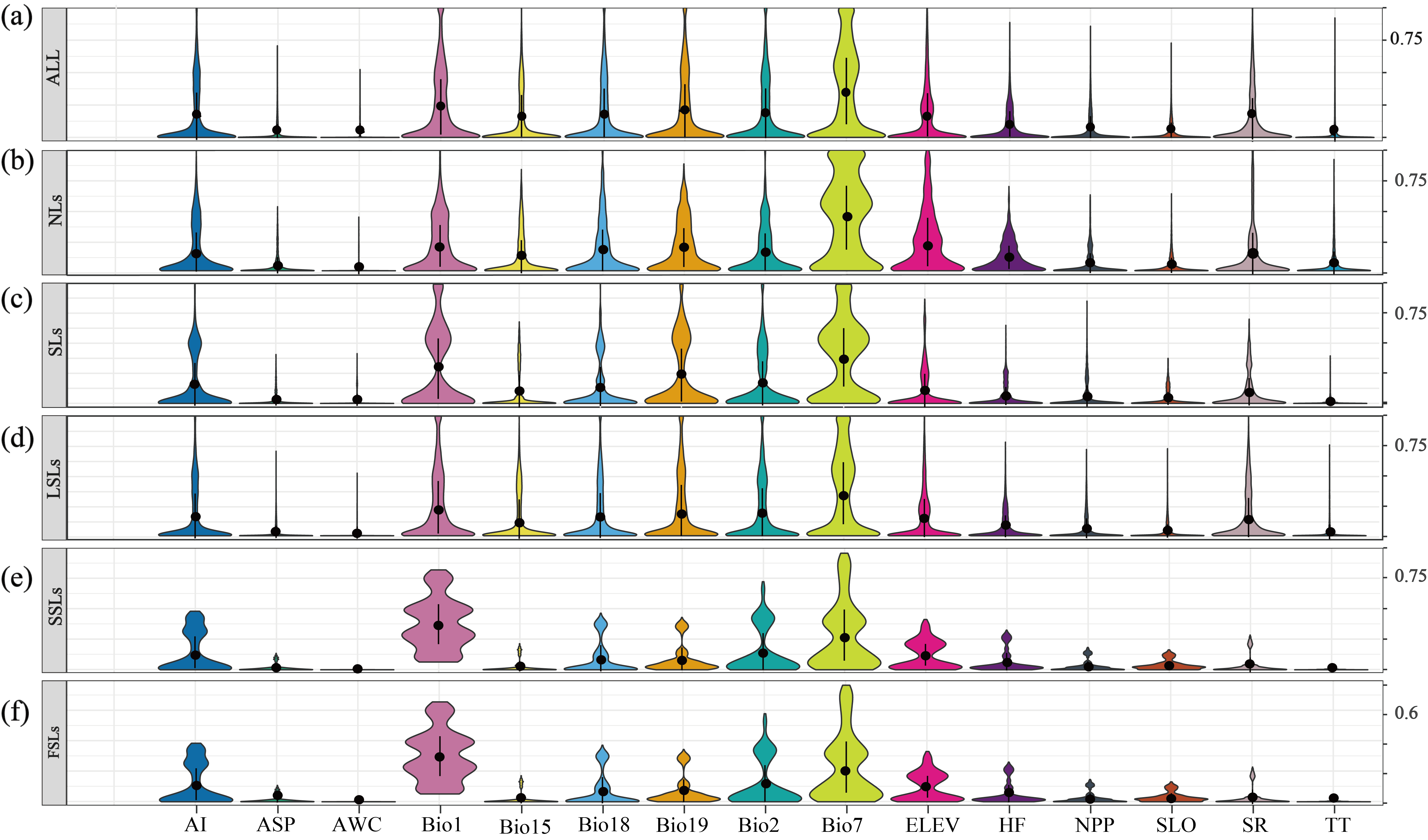


Figure. S4. Relative contributions of fifteen environmental variables as drivers to species richness of all plants (a) and other leaf-shaped gymnosperms (b-f). Needle leaves gymnosperms (NLs), scale leaves gymnosperms (SLs), lanceolate-shaped leaves gymnosperms (LSLs), fan-shaped leaves gymnosperms (FSLs), and strip-shaped leaves gymnosperms (SSLs). Bio1: Annual Mean Temperature; Bio2: mean diurnal range; Bio7: Temperature Annual Range; Bio15: precipitation seasonality; Bio18: Precipitation of Warmest Quarter; Bio19: Precipitation of Coldest Quarter; AI: aridity index; SR: solar radiation; AWC: available water capacity; EL: elevation; SLO: slope; ASP: aspect; NPP: net primary productivity; TT: topsoil texture; HFI: human footprint index.


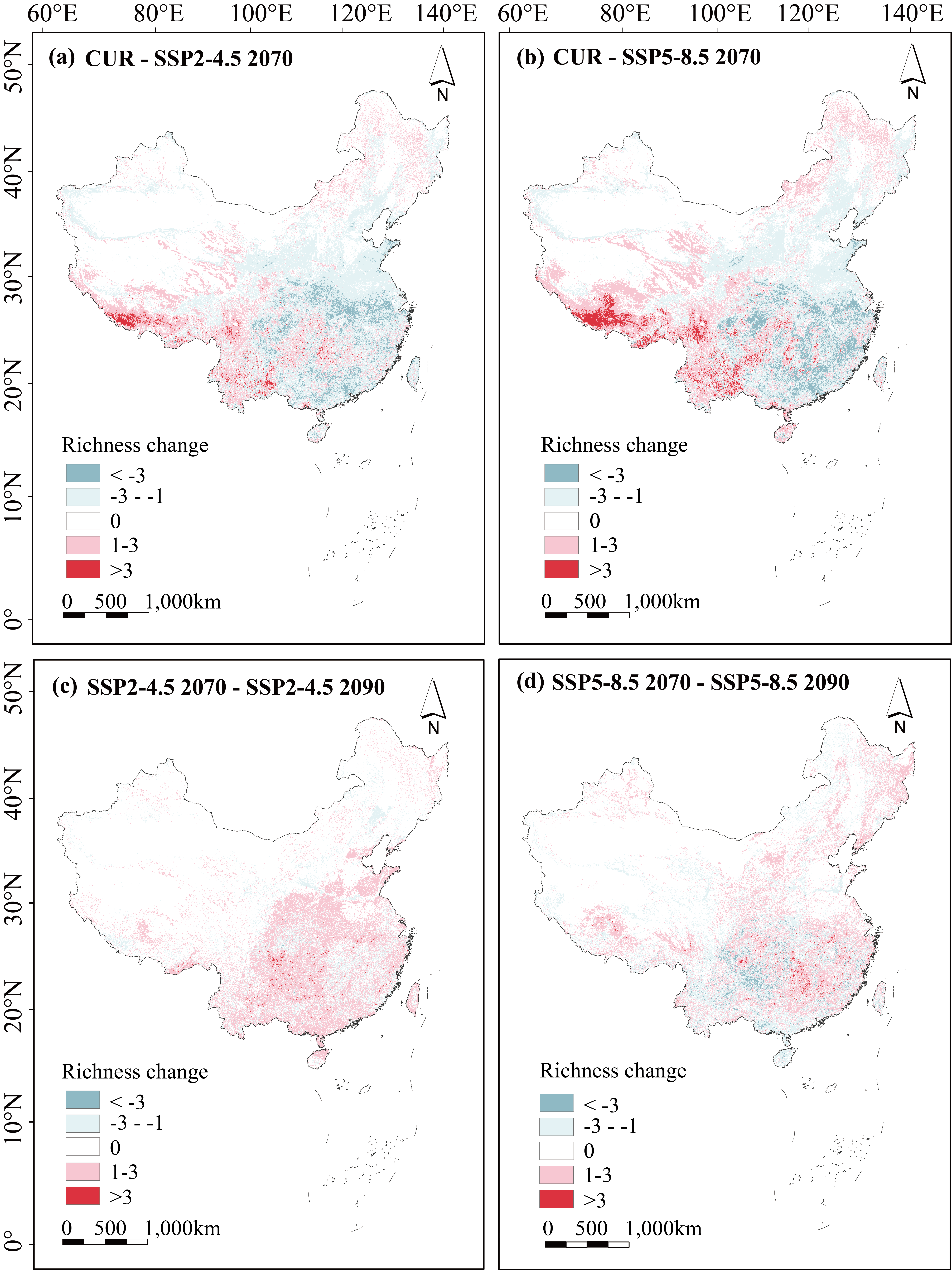


Figure. S5. The species quantity change distribution of gymnosperms under future climatic scenarios, from current to 2070, SSP2-4.5 (a), from current to 2070, SSP5-8.5 (b), from current to2090, SSP2-4.5 (c), from current to 2090, SSP5-8.5 (d).


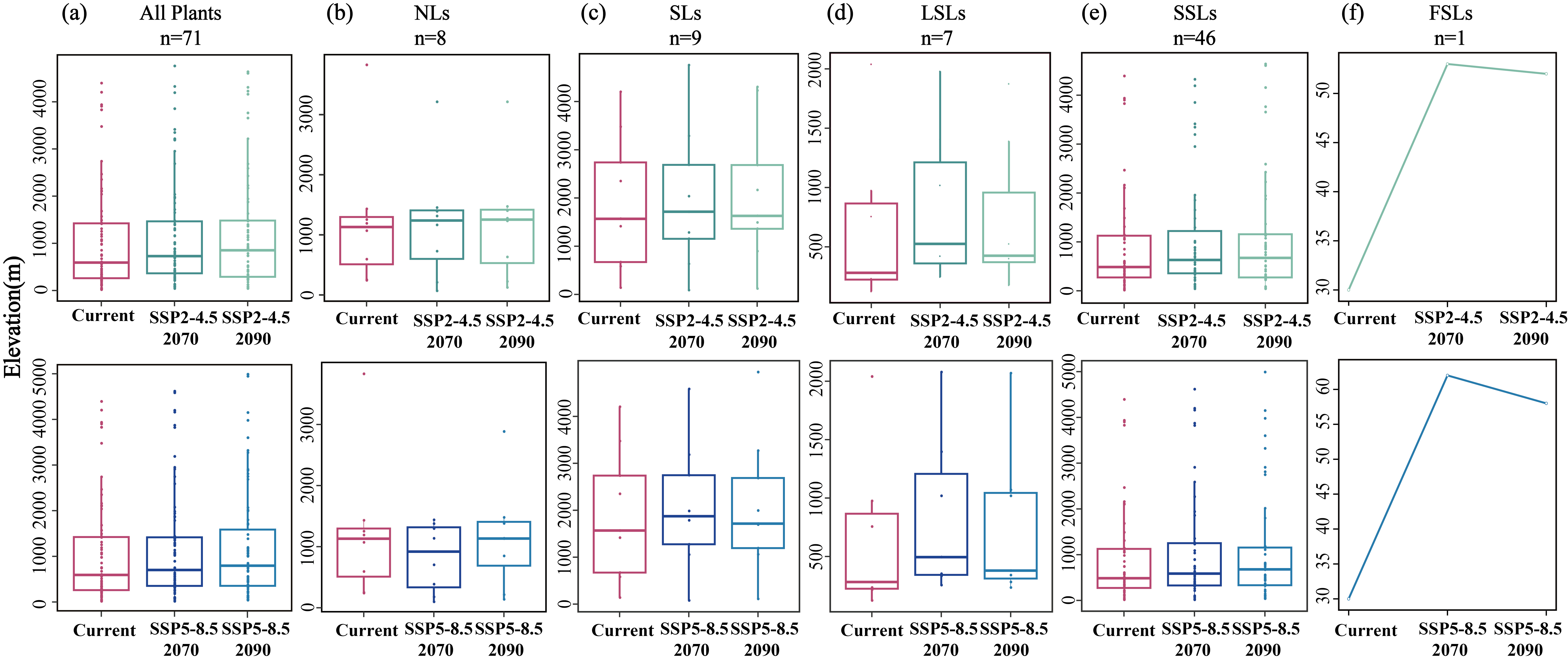


Figure. S6. Elevation centroid change trends for all plants (a) and other leaf-shaped gymnosperms (b-f) from the current period to 2090s. Needle leaves gymnosperms (NLs), scale leaves gymnosperms (SLs), lanceolate-shaped leaves gymnosperms (LSLs), fan-shaped leaves gymnosperms (FSLs), and strip-shaped leaves gymnosperms (SSLs).
