# Supplementary material for: Spatiotemporal Distribution Patterns and Conservation Priorities of Gymnosperms With Different Leaf Shapes in China Under Climate Change
Source: Ecol Evol. 2025 Aug 14;15(8):e71980. doi: 10.1002/ece3.71980 (PMC12354979; doi:10.1002/ece3.71980)
Supplement: Supplementary file 2 — Table S1: ece371980‐sup‐0002‐TablesS1‐S6.docx. [file ECE3-15-e71980-s001.docx]

**Table S1.** Species distribution modelling algorithms used to build ensemble model in this study.

| **Class of model** | **Method** | **Description of sub-model as used in Biomod2** |
| --- | --- | --- |
| Classification Tree Analysis | CTA | A decision tree model fitted with default settings in the underlying rpart package. Under biomod2 defaults it fits complex trees with many nodes. A five-fold internal cross-validation is used to choose the best model. |
| Flexible discriminant analysis | FDA | This method first fits a MARS model (fitted through mda package) then performs dimensionality reduction before attempting classification. |
| Generalized boosted regression models | GBM | A machine-learning method that ensembles regression trees through gradient boosting. A maximum of 2500 relatively deep trees are fitted, and best iteration of trees is selected using an internal three-fold cross-validation. |
| Generalized linear models | GLM | A regression model that fits quadratic response curves with no interactions between covariates, with stepwise backward selection using Akaike's information criterion. |
| Maximum entropy | MAXENT | It is a machine-learning method that estimates the species distribution probability by assessing the maximum entropy distribution, so that the most spread-out, or closest to uniform. |
| Random forests | RF | A machine-learning method that ensembles predictions from 500 classification trees, fitted on randomly selected subsets of all training data. Individual trees are controlled to have at least five data points in their terminal nodes, but are otherwise allowed to grow as many nodes as possible. |
| Surface Range Envelope | SRE | Modeling methods for determining the extent of species by comparing and identifying extremes in species distribution data and environmental variable data. |

**Table S2.** Percentage change in area and elevation centroid of for endangered gymnosperms in different periods.

| Time | Trend | Area change (percentage / No. of species) | | | |  |  | Elevation centroid change (percentage / No. of species) | | | |  |  |
| --- | --- | --- | --- | --- | --- | --- | --- | --- | --- | --- | --- | --- | --- |
|  |  | All plants (71) | NLs (8) | SLs (9) | LSLs (7) | SSLs (46) | FSLs(1) | All plants (71) | NLs (8) | SLs (9) | LSLs (7) | SSL(46) | FSL(1) |
| Current to 2070 ssp2-4.5 | Increase | 49.30% / 35 | 12.5% / 1 | 44.44% / 4 | 71.43% / 5 | 54.35% / 25 | 0% / 0 | 59.2% / 42 | 50% / 4 | 44.4% / 4 | 71.4% / 5 | 60.9% / 28 | 100%/1 |
|  | Decrease | 50.70% / 36 | 87.5% / 7 | 55.56% / 5 | 28.57% / 2 | 45.65% / 21 | 12.09% / 1 | 40.9% / 29 | 50% / 4 | 55.6% / 5 | 28.6% / 2 | 39.1% / 18 | 0% / 0 |
|  | Unchanged | 0% / 0 | 0% / 0 | 0% / 0 | 0% / 0 | 0% / 0 | 0% / 0 | 0% / 0 | 0% / 0 | 0% / 0 | 0% / 0 | 0% / 0 | 0% / 0 |
| Current to 2070 ssp5-8.5 | Increase | 46.48% / 33 | 25% / 2 | 44.44% / 4 | 57.15% / 4 | 50.00% / 23 | 0% / 0 | 61.9% / 44 | 37.5% / 3 | 55.6% / 5 | 100% / 7 | 58.7% / 27 | 100%/1 |
|  | Decrease | 53.52% / 38 | 75% / 6 | 55.56% / 5 | 42.86% / 3 | 50.00% / 23 | 22.14% / 1 | 38.1% / 27 | 62.5% / 5 | 44.4% / 4 | 0% / 0 | 41.3% / 19 | 0% / 0 |
|  | Unchanged | 0% / 0 | 0% / 0 | 0% / 0 | 0% / 0 | 0% / 0 | 0% / 0 | 0% / 0 | 0% / 0 | 0% / 0 | 0% / 0 | 0% / 0 | 0% / 0 |
| 2070 ssp2-4.5 to  2090 ssp2-4.5 | Increase | 45.07% / 32 | 37.5% / 3 | 22.22% / 2 | 71.43% / 5 | 47.83% / 22 | 0% / 0 | 45.1% / 32 | 62.5% / 5 | 66.7% / 6 | 14.3% / 1 | 43.5% / 20 | 0% / 0 |
|  | Decrease | 54.93% / 39 | 62.5% / 5 | 77.78% / 7 | 28.57% / 2 | 52.17% / 24 | 8.99% / 1 | 50.7% / 36 | 25.0% / 2 | 33.3% / 3 | 71.4% / 5 | 54.3% / 25 | 100%/1 |
|  | Unchanged | 0% / 0 | 0% / 0 | 0% / 0 | 0% / 0 | 0% / 0 | 0% / 0 | 4.2% / 3 | 12.5% / 1 | 0% / 0 | 14.3% / 1 | 2.2% / 1 | 0% / 0 |
| 2070 ssp5-8.5 to | Increase | 50.70% / 36 | 37.5% / 3 | 66.67% / 6 | 28.57% / 2 | 51.16% / 24 | 12.28% / 1 | 53.5% / 38 | 75.0% / 6 | 55.6% / 5 | 14.3% / 1 | 58.7% / 27 | 0% / 0 |
| 2090 ssp5-8.5 | Decrease | 49.30% / 35 | 62.5% / 5 | 33.33% / 3 | 71.43% / 5 | 48.84% / 22 | 0% / 0 | 45% / 32 | 25.0% / 2 | 44.4% / 4 | 71.4% / 5 | 41.3% / 19 | 100%/1 |
|  | Unchanged | 0% / 0 | 0% / 0 | 0% / 0 | 0% / 0 | 0% / 0 | 0% / 0 | 1.5% / 1 | 0% / 0 | 0% / 0 | 14.3% / 1 | 0% / 0 | 0% / 0 |

**Table S3.** The statistical results of the shifting amplitude of elevation and the shifting distance of the centroid of potential distributions for endangered gymnosperms in different periods, and their shifting velocities.

| **Time** | | **Shifting amplitude or distance** | | | **Shifting velocity per decade** | |
| --- | --- | --- | --- | --- | --- | --- |
|  |  | **Elevation (m)** | **The centroid of potential distributions (km^2^)** | **Elevation (m/10yr)** | | **The centroid of potential distributions (km^2^/10yr)** |
| **Current to 2070 ssp2-4.5** | 312.07 ± 410.19 | | 82.62 ± 82.24 | 36.71 ± 48.29 | | 9.72 ± 9.68 |
| **Current to** **2070 ssp5-8.5** | 377.09 ± 582.89 | | 120.43 ± 118.24 | 44.36 ± 68.58 | | 14.17 ± 13.91 |
| **2070 ssp2-4.5- to 2090 ssp2-4.5** | 223.70 ± 268.93 | | 16.27 ± 11.32 | 111.85 ± 134.46 | | 8.14 ± 5.66 |
| **2070 ssp5-8.5- to 2090 ssp5-8.5** | 163.25 ± 284.39 | | 36.59 ± 32.24 | 81.63 ± 142.20 | | 18.28 ± 16.12 |

**Table S4.** Average contribution rate of each environmental variable in models.

|  | **Bio1** | **Bio2** | **Bio7** | **Bio15** | **Bio18** | **Bio19** | **SRAD** | **AI** | **EL** | **SLO** | **ASP** | **NPP** | **AWC** | **TT** | **HFI** |
| --- | --- | --- | --- | --- | --- | --- | --- | --- | --- | --- | --- | --- | --- | --- | --- |
| All plants | 0.236 | 0.186 | 0.358 | 0.137 | 0.175 | 0.207 | 0.144 | 0.164 | 0.160 | 0.043 | 0.035 | 0.065 | 0.028 | 0.036 | 0.090 |
| NLs | 0.201 | 0.184 | 0.271 | 0.194 | 0.259 | 0.188 | 0.154 | 0.174 | 0.195 | 0.061 | 0.041 | 0.088 | 0.042 | 0.044 | 0.156 |
| SLs | 0.206 | 0.157 | 0.439 | 0.116 | 0.169 | 0.192 | 0.140 | 0.155 | 0.238 | 0.045 | 0.046 | 0.063 | 0.024 | 0.064 | 0.111 |
| LSLs | 0.292 | 0.168 | 0.386 | 0.100 | 0.143 | 0.238 | 0.098 | 0.160 | 0.109 | 0.042 | 0.031 | 0.063 | 0.030 | 0.014 | 0.062 |
| SSLs | 0.237 | 0.197 | 0.355 | 0.139 | 0.169 | 0.211 | 0.152 | 0.169 | 0.148 | 0.039 | 0.032 | 0.063 | 0.026 | 0.033 | 0.079 |
| FSLs | 0.325 | 0.131 | 0.247 | 0.026 | 0.083 | 0.079 | 0.036 | 0.125 | 0.107 | 0.033 | 0.016 | 0.028 | 0.001 | 0.002 | 0.058 |

*The average contribution of each variable of the combined model with the participation of the 15 environmental variables is shown in the table.

* Needle leaves gymnosperms (NLs), scale leaves gymnosperms (SLs), lanceolate-shaped leaves gymnosperms (LSLs), fan-shaped leaves gymnosperms (FSLs), and strip-shaped leaves gymnosperms (SSLs).

**Table S5.** Area change of endangered gymnosperms under the assumption of no migration for different climate scenarios.

| **Species** | **2070 ssp2-4.5** | **2070 ssp5-8.5** | **2090 ssp2-4.5** | **2090 ssp5-8.5** | **Species** | **2070 ssp2-4.5** | **2070 ssp5-8.5** | **2090 ssp2-4.5** | **2090 ssp5-8.5** |
| --- | --- | --- | --- | --- | --- | --- | --- | --- | --- |
| *Cycas micholitzii* | -16.06% | -14.90% | -14.92% | -16.65% | *Pinus dabeshanensis* | -50.19% | -63.08% | -53.02% | -48.18% |
| *Cycas pectinata* | -17.86% | -23.93% | -19.69% | -14.62% | *Pinus koraiensis* | -22.14% | -39.50% | -22.05% | -15.08% |
| *Cycas hainanensis* | -2.80% | -3.79% | -0.90% | -11.91% | *Pinus kwangtungensis* | -12.94% | -25.09% | -17.91% | -43.92% |
| *Cycas szechuanensis* | -15.56% | -23.62% | -15.99% | -17.77% | *Pseudolarix amabilis* | -25.59% | -41.13% | -25.48% | -37.36% |
| *Cycas taiwaniana* | 0.00% | 0.00% | 0.17% | 0.17% | *Pseudotsuga forrestii* | -17.69% | -27.15% | -20.14% | -20.77% |
| *Cycas revoluta* | -14.20% | -25.48% | -17.55% | -17.57% | *Pseudotsuga brevifolia* | -3.67% | -5.48% | -3.72% | -10.63% |
| *Cycas guizhouensis* | -13.23% | -17.76% | -22.65% | -18.33% | *Pseudotsuga sinensis* | -30.34% | -49.27% | -36.43% | -37.50% |
| *Cycas debaoensis* | -4.22% | -2.05% | -1.46% | -1.86% | *Pseudotsuga gaussenii* | -12.17% | -24.76% | -8.64% | -23.86% |
| *Ginkgo biloba* | -39.03% | -59.64% | -45.49% | -41.33% | *Pinus henryi* | -7.36% | -6.65% | -8.16% | -11.33% |
| *Podocarpus fleuryi* | 0.00% | 0.00% | 0.14% | 0.14% | *Pinus sylvestris*var.*mongolica* | -7.04% | -12.90% | -8.05% | -16.66% |
| *Podocarpus imbricatus* | -0.68% | -0.53% | -1.05% | -0.59% | *Picea likiangensis* var. *linzhiensis* | -8.50% | -12.82% | -10.37% | -9.25% |
| *Podocarpus macrophyllus* | -11.24% | -23.39% | -13.49% | -23.02% | *Picea brachytyla* var. *complanata* | -9.34% | -13.57% | -11.28% | -11.91% |
| *Nageia nagi* | -2.40% | -4.49% | -2.35% | -3.56% | *Picea brachytyla* | -16.08% | -25.78% | -20.24% | -19.06% |
| *Podocarpus neriifolius* | -0.76% | -1.85% | -1.59% | -2.21% | *Picea meyeri* | -33.47% | -51.32% | -38.61% | -36.68% |
| *Cephalotaxus hainanensis* | -6.01% | -7.10% | -5.10% | -9.05% | *Larix speciosa* | -26.30% | -34.91% | -28.91% | -28.56% |
| *Cephalotaxus oliveri* | -1.91% | -2.96% | -2.52% | -4.58% | *Larix gmelinii* var. *principis-rupprechtii* | -19.82% | -27.00% | -22.28% | -30.43% |
| *Cephalotaxus sinensis* | -20.18% | -34.81% | -24.94% | -28.50% | *Abies georgei* | -15.95% | -26.70% | -19.41% | -18.50% |
| *Cephalotaxus mannii* | -0.78% | -3.97% | -2.72% | -5.49% | *Abies recurvata* | -80.58% | -80.33% | -80.65% | -80.51% |
| *Cephalotaxus latifolia* | -8.77% | -16.79% | -15.19% | -19.79% | *Abies squamata* | -1.14% | -1.53% | -1.19% | -2.24% |
| *Amentotaxus argotaenia* | -1.99% | -3.62% | -2.50% | -5.22% | *Tsuga forrestii* | -6.44% | -15.86% | -8.14% | -10.07% |
| *Amentotaxus yunnanensis* | -17.40% | -21.72% | -18.78% | -21.43% | *Calocedrus macrolepis* | -2.66% | -5.76% | -5.04% | -6.71% |
| *Pseudotaxus chienii* | -0.34% | -0.54% | -0.12% | -2.59% | *Cupressus chengiana* | -7.14% | -7.47% | -7.27% | -8.07% |
| *Taxus cuspidata* | -14.33% | -20.64% | -13.42% | -5.09% | *Cupressus torulosa* | -15.42% | -22.11% | -18.26% | -21.28% |
| *Taxus wallichiana* | -0.18% | -0.18% | -0.07% | -0.39% | *Chamaecyparis hodginsii* | -10.81% | -29.11% | -17.09% | -27.17% |
| *Taxus wallichiana* var. *chinensis* | -21.77% | -38.48% | -27.47% | -22.31% | *Metasequoia glyptostroboides* | -49.61% | -73.56% | -61.35% | -63.83% |
| *Taxus wallichiana* var. *mairei* | -2.60% | -5.64% | -2.50% | -5.52% | *Taiwania cryptomerioides* | -47.31% | -62.96% | -50.17% | -53.07% |
| *Torreya fargesii* | -21.24% | -23.47% | -20.35% | -21.89% | *Glyptostrobus pensilis* | -0.19% | -0.64% | -0.27% | -0.24% |
| *Torreya grandis* | -39.88% | -62.57% | -35.26% | -41.49% | *Juniperus tibetica* | -1.52% | -3.10% | -2.04% | -1.87% |
| *Torreya jackii* | -9.94% | -17.11% | -5.66% | -19.32% | *Juniperus rigida* | -1.09% | -0.77% | -0.65% | -4.23% |
| *Torreya yunnanensis* | -2.50% | -2.59% | -2.06% | -2.92% | *Cupressus duclouxiana* | -7.72% | -8.51% | -7.93% | -6.68% |
| *Abies chensiensis* | -40.45% | -50.18% | -42.71% | -41.40% | *Ephedra intermedia* | -18.44% | -24.35% | -20.96% | -14.57% |
| *Cathaya argyrophylla* | -51.00% | -71.80% | -59.32% | -75.15% | *Ephedra sinica* | -34.69% | -53.59% | -42.08% | -43.51% |
| *Keteleeria davidiana* var. *calcarea* | -16.04% | -26.99% | -19.35% | -23.90% | *Gnetum montanum* | -1.39% | -2.17% | -1.18% | -1.44% |
| *Keteleeria fortunei* var. *cyclolepis* | -6.15% | -9.13% | -8.25% | -9.08% | *Gnetum parvifolium* | -0.42% | -0.94% | -1.09% | -1.46% |
| *Keteleeria pubescens* | -0.80% | -3.89% | -3.14% | -6.61% | *Gnetum pendulum* | -5.34% | -3.83% | -4.84% | -47.02% |
| *Picea neoveitchii* | -23.70% | -18.36% | -22.51% | -21.55% |  |  |  |  |  |

* According to the IUCN Red List assessment criteria A3(c), the percentage loss of a species' current potential range corresponds to its threat level as follows: 100% indicates Extinct (EX), 80% indicates Critically Endangered (CR), 50% indicates Endangered (EN), 30% indicates Vulnerable (VU), 10% indicates Near Threatened (NT), and less than 10% indicates Least Concern (LC).

**Table S6.** Area change of endangered gymnosperms under the assumption of unlimited migration for different climate scenarios.

| **Species** | **2070 ssp2-4.5** | **2070 ssp5-8.5** | **2090 ssp2-4.5** | **2090 ssp5-8.5** | **Species** | **2070 ssp2-4.5** | **2070 ssp5-8.5** | **2090 ssp2-4.5** | **2090 ssp5-8.5** |
| --- | --- | --- | --- | --- | --- | --- | --- | --- | --- |
| *Cycas micholitzii* | -10.58% | -4.27% | -6.54% | -8.26% | *Pinus dabeshanensis* | -45.88% | -58.51% | -47.83% | -40.04% |
| *Cycas pectinata* | 37.63% | 66.66% | 50.52% | 71.90% | *Pinus koraiensis* | -21.49% | -38.19% | -20.94% | -4.15% |
| *Cycas hainanensis* | 26.37% | 26.47% | 34.65% | 3.26% | *Pinus kwangtungensis* | -10.97% | -23.35% | -15.98% | -42.43% |
| *Cycas szechuanensis* | 38.77% | 27.52% | 38.44% | 40.56% | *Pseudolarix amabilis* | -24.35% | -40.62% | -24.78% | -36.14% |
| *Cycas taiwaniana* | 115.12% | 210.40% | 134.46% | 143.66% | *Pseudotsuga forrestii* | -4.17% | -7.05% | -7.63% | 1.22% |
| *Cycas revoluta* | 18.85% | 23.33% | 18.20% | 19.42% | *Pseudotsuga brevifolia* | 15.90% | 16.59% | 19.30% | 2.33% |
| *Cycas guizhouensis* | 8.32% | 7.01% | 1.61% | 9.62% | *Pseudotsuga sinensis* | -14.86% | -31.30% | -20.93% | -20.75% |
| *Cycas debaoensis* | 28.72% | 49.83% | 47.58% | 41.39% | *Pseudotsuga gaussenii* | -11.44% | -24.36% | -6.50% | -23.00% |
| *Ginkgo biloba* | -32.20% | -52.81% | -40.03% | -29.59% | *Pinus henryi* | -6.86% | -3.97% | -5.62% | -7.31% |
| *Podocarpus fleuryi* | 109.80% | 154.81% | 127.08% | 124.14% | *Pinus sylvestris*var.*mongolica* | -6.06% | -12.34% | -7.46% | -16.22% |
| *Podocarpus imbricatus* | 38.80% | 55.45% | 42.49% | 44.18% | *Picea likiangensis* var. *linzhiensis* | 66.87% | 97.35% | 68.22% | 115.76% |
| *Podocarpus macrophyllus* | -10.88% | -23.25% | -13.14% | -22.72% | *Picea brachytyla* var. *complanata* | 5.09% | 5.58% | 0.05% | 17.82% |
| *Nageia nagi* | 1.44% | -1.38% | 2.69% | 2.18% | *Picea brachytyla* | -5.95% | -12.27% | -10.41% | -8.85% |
| *Podocarpus neriifolius* | 19.05% | 25.78% | 20.42% | 24.59% | *Picea meyeri* | -33.46% | -51.32% | -38.61% | -36.48% |
| *Cephalotaxus hainanensis* | 4.78% | 12.22% | 14.12% | 10.87% | *Larix speciosa* | -4.20% | -0.01% | -8.19% | 10.44% |
| *Cephalotaxus oliveri* | 0.00% | -0.59% | -0.72% | -0.92% | *Larix gmelinii* var. *principis-rupprechtii* | -19.05% | -26.18% | -21.46% | -29.58% |
| *Cephalotaxus sinensis* | -19.14% | -33.80% | -24.13% | -27.76% | *Abies georgei* | 18.17% | 27.00% | 15.16% | 38.71% |
| *Cephalotaxus mannii* | 1.08% | -2.96% | -1.57% | -3.92% | *Abies recurvata* | -80.58% | -80.33% | -80.65% | -80.51% |
| *Cephalotaxus latifolia* | -3.00% | -11.54% | -11.21% | -12.92% | *Abies squamata* | 33.77% | 47.22% | 37.65% | 41.20% |
| *Amentotaxus argotaenia* | -0.99% | -2.81% | -0.75% | -3.70% | *Tsuga forrestii* | 4.75% | 0.82% | 4.31% | 5.10% |
| *Amentotaxus yunnanensis* | -14.39% | -17.98% | -16.34% | -15.32% | *Calocedrus macrolepis* | 10.73% | 8.94% | 6.15% | 5.36% |
| *Pseudotaxus chienii* | 8.96% | 10.26% | 15.23% | 5.65% | *Cupressus chengiana* | 1.94% | 6.28% | 2.98% | 5.79% |
| *Taxus cuspidata* | -13.31% | -18.41% | -11.93% | 19.73% | *Cupressus torulosa* | 12.49% | 27.95% | 11.06% | 38.07% |
| *Taxus wallichiana* | 48.36% | 77.25% | 55.78% | 65.40% | *Chamaecyparis hodginsii* | -9.02% | -28.65% | -15.98% | -25.59% |
| *Taxus wallichiana* var. *chinensis* | -21.57% | -38.26% | -27.26% | -21.45% | *Metasequoia glyptostroboides* | -49.38% | -73.48% | -61.28% | -63.56% |
| *Taxus wallichiana* var. *mairei* | -0.15% | -3.73% | 0.30% | -0.27% | *Taiwania cryptomerioides* | -31.52% | -51.39% | -34.66% | -37.98% |
| *Torreya fargesii* | -19.57% | -19.56% | -17.93% | -16.63% | *Glyptostrobus pensilis* | 3.71% | 2.73% | 4.59% | 5.64% |
| *Torreya grandis* | -39.09% | -61.52% | -33.83% | -38.69% | *Juniperus tibetica* | 70.52% | 99.18% | 79.68% | 85.17% |
| *Torreya jackii* | -6.45% | -13.96% | 3.25% | -11.34% | *Juniperus rigida* | 25.65% | 40.69% | 31.49% | 17.31% |
| *Torreya yunnanensis* | 14.83% | 23.90% | 19.11% | 19.72% | *Cupressus duclouxiana* | -4.29% | -2.84% | -4.89% | 2.66% |
| *Abies chensiensis* | -39.97% | -49.59% | -42.12% | -38.62% | *Ephedra intermedia* | -5.70% | -8.93% | -8.26% | 17.68% |
| *Cathaya argyrophylla* | -50.48% | -71.37% | -58.92% | -74.81% | *Ephedra sinica* | -33.73% | -51.79% | -41.16% | -40.12% |
| *Keteleeria davidiana* var. *calcarea* | 72.55% | 67.40% | 71.67% | 66.07% | *Gnetum montanum* | 34.26% | 48.02% | 42.00% | 38.09% |
| *Keteleeria fortunei* var. *cyclolepis* | 22.92% | 22.69% | 22.29% | 19.46% | *Gnetum parvifolium* | 12.15% | 15.43% | 10.30% | 9.17% |
| *Keteleeria pubescens* | 8.19% | 1.18% | 2.40% | -1.95% | *Gnetum pendulum* | 2.97% | 8.77% | 6.80% | -47.01% |
| *Picea neoveitchii* | -18.24% | -0.97% | -13.70% | -8.66% |  |  |  |  |  |

* According to the IUCN Red List assessment criteria A3(c), the percentage loss of a species' current potential range corresponds to its threat level as follows: 100% indicates Extinct (EX), 80% indicates Critically Endangered (CR), 50% indicates Endangered (EN), 30% indicates Vulnerable (VU), 10% indicates Near Threatened (NT), and less than 10% indicates Least Concern (LC).
